# Supplementary material for: A multi-agent approach to neurological clinical reasoning
Source: PLOS Digit Health. 2025 Dec 4;4(12):e0001106. doi: 10.1371/journal.pdig.0001106 (PMC12677565; doi:10.1371/journal.pdig.0001106)
Supplement: S1 File — Table A. Comparison of LLMs and RLMs specifications and deployment characteristics. Table B. Distribution of neurology subspecialties across different exams. Table C. Accuracy analysis of base, RAG enhanced, and agentic methods across neurological subspecialties. Table D. Comparative performance on board certification (N = 305) and MedQA neurological questions (N = 155). Table E. Accuracy analysis of base, RAG enhanced, and agentic methods across neurological subspecialties in exam 1062023. Table F. Accuracy analysis of base, RAG enhanced, and agentic methods across neurological subspecialties in exam 1052024. Table G. Accuracy analysis of base, RAG Enhanced, and agentic methods across neurological subspecialties in exam 1092024. (DOCX) [file pdig.0001106.s001.docx]

S1 File.

**Table A.**  Comparison of LLMs and RLMs Specifications and Deployment Characteristics.

| **Model** | **Domain** | **Parameters** | **Context Window** | **Max Output**  **Tokens** | **Deployment** | **Vendor** |
| --- | --- | --- | --- | --- | --- | --- |
| o1 | General | ∼2T* | 200K | 100K | API | OpenAI |
| GPT-4o | General | ∼1.8T* | 128K | 16K | API | OpenAI |
| GPT-4o-mini | General | ∼1.5T* | 128K | 16K | API | OpenAI |
| LLaMA 3.3-70B | General | 70B | 128K | 4K | Ollama | Meta |
| LLaMA 3.1-8B | General | 8B | 128K | 2K | Ollama | Meta |
| DeepSeek-R1-70B | General | 70B | 128K | 4K | Ollama | DeepSeek |
| DeepSeek-R1-8B | General | 8B | 128K | 4K | Ollama | DeepSeek |
| OpenBioLLM-70B | Medical | 70B | 128K | 4K | Ollama | Saama |
| Medllama3 V20 | Medical | 8B | 128K | 2K | Ollama | Probe Medical |
| Meditron-70B | Medical | 70B | 128K | 2K | Ollama | EPFLS/Yale |

* Parameter counts are approximated for proprietary models. Context and output token limits reflect maximum values used in our experiments.

**Table B. Distribution of Neurology Subspecialties Across Different Exams.**

| **Exam**  **Subspecialties** | **1062023**  **(N=134)** | **1052024**  **(N=85)** | **1092024**  **(N=86)** | **All**  **(N=305)** |
| --- | --- | --- | --- | --- |
| Behavioral & Cognitive Neurology | 11.90% | 15.30% | 19.80% | 15.08% |
| CSF Circulation Disorders | 5.20% | 1.20% | 1.20% | 2.95% |
| Epilepsy | 1.50% | 4.70% | 2.30% | 2.62% |
| Genetic Neurology | 9.70% | 8.20% | 7.00% | 8.52% |
| Headache and Dizziness | 0.00% | 1.20% | 3.50% | 1.31% |
| Infectious Neurology | 6.70% | 3.50% | 2.30% | 4.59% |
| Miscellaneous | 3.70% | 4.70% | 4.70% | 4.26% |
| Movement Disorders | 10.50% | 11.80% | 8.10% | 10.16% |
| Neuro-oncology | 9.00% | 8.20% | 4.70% | 7.54% |
| Neuroimmunology | 3.70% | 9.40% | 9.30% | 6.89% |
| Neuromuscular | 24.60% | 20.00% | 19.80% | 21.97% |
| Neurophthalmology | 4.50% | 7.10% | 7.00% | 5.90% |
| Vascular Neurology | *9.00%* | *4.70%* | *10.50%* | 8.20% |
|  |  |  |  |  |

**Table C. Accuracy Analysis of Base, RAG Enhanced, and Agentic Methods Across Neurological Subspecialties.**

| **MODEL** | **METHOD** | Behavioral &Neurology (N=46) | CSF Disorders  (N=9) | Epilepsy (N=8) | Genetic (N=26) | Headache & Dizziness  (N=4) | Infectious (N=14) | Miscellaneous (N=13) | Movement (N=31) | Neuro-oncology (N=23) | Neuro-immunology (N=21) | Neuro-muscular (N=67) | Neuro-phthalmology (N=18) | Vascular Neurology (N=25) |
| --- | --- | --- | --- | --- | --- | --- | --- | --- | --- | --- | --- | --- | --- | --- |
| o1 | Agentic | 100% | 100% | 100% | 85% | 100% | 100% | 100% | 87% | 100% | 100% | 94% | 94% | 92% |
|  | RAG | 100% | 100% | 88% | 92% | 100% | 93% | 92% | 87% | 96% | 100% | 88% | 89% | 92% |
|  | Base | 96% | 100% | 100% | 85% | 100% | 86% | 100% | 84% | 96% | 95% | 91% | 89% | 88% |
| GPT-4o | Agentic | 89% | 89% | 88% | 85% | 100% | 100% | 92% | 84% | 100% | 95% | 88% | 89% | 88% |
|  | RAG | 89% | 89% | 88% | 92% | 100% | 93% | 92% | 84% | 87% | 91% | 82% | 94% | 80% |
|  | Base | 87% | 89% | 75% | 81% | 75% | 100% | 85% | 81% | 91% | 86% | 76% | 67% | 72% |
| LLaMA 3.3-70B | Agentic | 87% | 88% | 88% | 81% | 100% | 100% | 85% | 94% | 100% | 86% | 93% | 89% | 76% |
|  | RAG | 78% | 89% | 75% | 73% | 50% | 79% | 77% | 81% | 74% | 67% | 72% | 72% | 68% |
|  | Base | 83% | 44% | 75% | 77% | 100% | 86% | 77% | 68% | 78% | 67% | 63% | 44% | 64% |
| DeepSeek-R1-70B | RAG | 91% | 89% | 75% | 81% | 100% | 86% | 100% | 84% | 96% | 81% | 85% | 94% | 88% |
|  | Base | 91% | 100% | 88% | 77% | 75% | 86% | 100% | 84% | 96% | 81% | 88% | 82% | 88% |
| GPT-4o-mini | RAG | 72% | 78% | 88% | 77% | 100% | 71% | 92% | 74% | 70% | 81% | 75% | 44% | 72% |
|  | Base | 70% | 56% | 75% | 50% | 100% | 57% | 92% | 55% | 70% | 48% | 60% | 50% | 60% |
| OpenBioLLM-70B | RAG | 70% | 78% | 75% | 77% | 75% | 79% | 69% | 68% | 83% | 67% | 63% | 67% | 64% |
|  | Base | 76% | 56% | 50% | 69% | 100% | 79% | 69% | 71% | 70% | 71% | 64% | 56% | 44% |
| DeepSeek-R1-8B | RAG | 63% | 78% | 75% | 62% | 100% | 86% | 77% | 77% | 70% | 67% | 66% | 61% | 60% |
|  | Base | 48% | 56% | 38% | 46% | 75% | 57% | 46% | 55% | 52% | 43% | 45% | 22% | 44% |
| LLaMA 3.1-8B | RAG | 72% | 67% | 50% | 62% | 100% | 86% | 69% | 68% | 65% | 43% | 69% | 67% | 56% |
|  | Base | 70% | 33% | 63% | 54% | 75% | 57% | 69% | 55% | 48% | 52% | 63% | 61% | 44% |
| Medllama3-V20 | RAG | 57% | 56% | 50% | 50% | 75% | 57% | 54% | 58% | 65% | 43% | 63% | 39% | 44% |
|  | Base | 61% | 44% | 38% | 46% | 50% | 21% | 31% | 42% | 52% | 33% | 57% | 44% | 32% |
| Meditron-70B | RAG | 46% | 56% | 25% | 46% | 25% | 43% | 69% | 32% | 39% | 62% | 34% | 39% | 36% |
|  | Base | 59% | 44% | 25% | 46% | 25% | 86% | 69% | 32% | 61% | 62% | 66% | 39% | 32% |

This table presents the accuracy percentages tested on 13 neurological subspecialties. Each subspecialty column indicates the number of test questions (N) in parentheses. Performance is reported as percentage accuracy, displaying the comparative effectiveness of each method across different neurological domains.

**Table D. Comparative Performance on Board Certification (N=305) and MedQA Neurological Questions (N=155).**

| Model | Method | Board Certification | | MedQA Neurology | | | | Performance | |  |
| --- | --- | --- | --- | --- | --- | --- | --- | --- | --- | --- |
|  |  | **Accuracy (95% CI)** | **F1** | | **Accuracy (95% CI)** | **F1** | **Difference** | | F1 Diff. | |
| o1 | Base | 90.9% (87.1-93.6) | 0.952 | | 96.8% (92.7-98.6) | 0.984 | +5.9% | | +0.032 | |
|  | RAG | 92.2% (88.6-94.7) | 0.959 | | 94.8% (89.3-96.9) | 0.974 | +2.6% | | +0.015 | |
|  | Agents | 94.6% (91.3-96.5) | 0.973 | | 94.8% (89.3-96.9) | 0.974 | +0.2% | | +0.001 | |
| GPT-4o | Base | 80.5% (75.5-84.4) | 0.892 | | 85.2% (78.7-89.9) | 0.920 | +4.7% | | +0.028 | |
|  | RAG | 87.3% (83.0-90.5) | 0.932 | | 89.7% (83.9-93.5) | 0.946 | +2.4% | | +0.014 | |
|  | Agents | 89.3% (85.2-92.2) | 0.943 | | 94.8% (89.3-96.9) | 0.974 | +5.5% | | +0.031 | |
| LLaMA 3.3-70B | Base | 69.5% (63.8-74.1) | 0.820 | | 76.8% (69.5-82.7) | 0.869 | +7.3% | | +0.049 | |
|  | RAG | 73.4% (67.9-77.8) | 0.846 | | 74.8% (66.8-80.4) | 0.856 | +1.4% | | +0.010 | |
|  | Agents | 89.2% (85.2-92.2) | 0.943 | | 81.3% (74.4-86.6) | 0.897 | −7.9% | | −0.046 | |

This table presents the performance metrics of various language models on two distinct datasets: Board Certification questions and the neurological subset of MedQA questions. The difference column highlights the performance gap between the two datasets.

**Table E. Accuracy Analysis of Base, RAG Enhanced, and Agentic Methods Across Neurological Subspecialties in Exam 1062023.**

| **Subspecialty** | **o1** | | | **GPT-4o** | | | **LLaMA 3.3-70B** | | |
| --- | --- | --- | --- | --- | --- | --- | --- | --- | --- |
|  | **Base** | **RAG** | **Agents** | **Base** | **RAG** | **Agents** | **Base** | **RAG** | **Agents** |
| Behavioral & Cognitive Neurology | 93.75% | 100% | 93.33% | 87.5% | 81.25% | 87.5% | 87.5% | 87.5% | 93.75% |
| CSF Circulation Disorders | 100% | 100% | 85.71% | 100% | 100% | 85.71% | 42.86% | 85.71% | 100% |
| Epilepsy | 100% | 100% | 100% | 100% | 100% | 100% | 100% | 100% | 100% |
| Genetic Neurology | 76.92% | 92.31% | 91.67% | 84.62% | 84.62% | 84.62% | 76.92% | 69.23% | 100% |
| Infectious Neurology | 88.89% | 88.89% | 87.5% | 100% | 88.89% | 100% | 77.78% | 66.67% | 100% |
| Miscellaneous | 100% | 100% | 75% | 75% | 100% | 100% | 60% | 80% | 75% |
| Movement Disorders | 85.71% | 85.71% | 100% | 78.57% | 85.71% | 71.43% | 78.57% | 78.57% | 85.71% |
| Neuro-oncology | 100% | 100% | 100% | 91.67% | 83.33% | 100% | 83.33% | 75% | 90.91% |
| Neuroimmunology | 100% | 100% | 60% | 80% | 100% | 80% | 80% | 80% | 100% |
| Neuromuscular | 90.91% | 84.85% | 96.77% | 72.73% | 81.82% | 90.91% | 63.64% | 69.7% | 84.85% |
| Neurophthalmology | 83.33% | 83.33% | 100% | 83.33% | 83.33% | 83.33% | 50% | 66.67% | 83.33% |
| Vascular Neurology | 75% | 91.67% | 90.91% | 75% | 66.67% | 100% | 50% | 58.33% | 81.82% |

This table presents the accuracy percentages of three language models (O1, GPT-4o, and LLaMA 3.3-70B) across Base, RAG- Enhanced, and Agentic methods and for each neurological subspecialties for exam 1062023.

**Table F. Accuracy Analysis of Base, RAG Enhanced, and Agentic Methods Across Neurological Subspecialties in Exam 1052024.**

| **Subspecialty** | **o1** | | | **GPT-4o** | | | **LLaMA 3.3-70B** | | |
| --- | --- | --- | --- | --- | --- | --- | --- | --- | --- |
|  | **Base** | **RAG** | **Agents** | **Base** | **RAG** | **Agents** | **Base** | **RAG** | **Agents** |
| Behavioral & Cognitive Neurology | 100% | 100% | 100% | 84.62% | 92.31% | 84.62% | 76.92% | 76.92% | 76.92% |
| CSF Circulation Disorders | 100% | 100% | 100% | 100% | 0% | 100% | 100% | 100% | 100% |
| Epilepsy | 100% | 100% | 100% | 100% | 100% | 100% | 50% | 75% | 100% |
| Genetic Neurology | 85.71% | 85.71% | 85.71% | 71.43% | 100% | 71.43% | 100% | 85.71% | 100% |
| Headache and Dizziness | 100% | 100% | 100% | 100% | 100% | 100% | 100% | 100% | 100% |
| Infectious Neurology | 66.67% | 100% | 100% | 100% | 100% | 100% | 100% | 100% | 100% |
| Miscellaneous | 100% | 75% | 100% | 75% | 75% | 75% | 100% | 50% | 75% |
| Movement Disorders | 70% | 80% | 70% | 70% | 80% | 90% | 50% | 70% | 90% |
| Neuro-oncology | 85.71% | 85.71% | 100% | 85.71% | 85.71% | 100% | 71.43% | 85.71% | 100% |
| Neuroimmunology | 87.5% | 100% | 100% | 87.5% | 87.5% | 100% | 50% | 50% | 87.5% |
| Neuromuscular | 88.24% | 94.12% | 94.12% | 70.59% | 82.35% | 88.24% | 70.59% | 76.47% | 94.12% |
| Neurophthalmology | 100% | 100% | 100% | 50% | 100% | 83.33% | 33.33% | 66.67% | 83.33% |
| Vascular Neurology | 100% | 75% | 100% | 100% | 75% | 75% | 50% | 75% | 50% |

This table presents the accuracy percentages of three language models (O1, GPT-4o, and LLaMA 3.3-70B) across Base, RAG- Enhanced, and Agentic methods and for each neurological subspecialties for exam 1062023.

**Table G. Accuracy Analysis of Base, RAG Enhanced, and Agentic Methods Across Neurological Subspecialties in Exam 1092024.**

|  | **o1** | | | **GPT-4o** | | | **LLaMA 3.3-70B** | | |
| --- | --- | --- | --- | --- | --- | --- | --- | --- | --- |
| **Subspecialty** | **Base** | **RAG** | **Agents** | **Base** | **RAG** | **Agents** | **Base** | **RAG** | **Agents** |
| Behavioral & Cognitive Neurology | 88.24% | 82.35% | 94.12% | 94.12% | 70.59% | 100% | 94.12% | 94.12% | 94.12% |
| CSF Circulation Disorders | 0% | 0% | 100% | 100% | 100% | 100% | 100% | 100% | 100% |
| Epilepsy | 0% | 100% | 100% | 50% | 50% | 50% | 50% | 100% | 100% |
| Genetic Neurology | 83.33% | 50% | 100% | 100% | 66.67% | 100% | 100% | 83.33% | 100% |
| Headache and Dizziness | 66.67% | 100% | 100% | 100% | 33.33% | 100% | 100% | 100% | 100% |
| Infectious Neurology | 100% | 100% | 100% | 100% | 100% | 100% | 100% | 50% | 100% |
| Miscellaneous | 100% | 75% | 100% | 100% | 100% | 100% | 100% | 50% | 100% |
| Movement Disorders | 100% | 71.43% | 100% | 85.71% | 100% | 100% | 100% | 100% | 100% |
| Neuro-oncology | 100% | 75% | 100% | 100% | 50% | 100% | 100% | 100% | 100% |
| Neuroimmunology | 87.50% | 75% | 100% | 87.50% | 75% | 100% | 100% | 75% | 100% |
| Neuromuscular | 88.24% | 52.94% | 94.12% | 82.35% | 70.59% | 88.24% | 82.35% | 94.12% | 100% |
| Neurophthalmology | 66.67% | 50% | 83.33% | 100% | 83.33% | 83.33% | 100% | 100% | 100% |
| Vascular Neurology | 50% | 88.89% | 100% | 100% | 77.78% | 100% | 77.78% | 77.78% | 100% |

This table presents the accuracy percentages of three language models (O1, GPT-4o, and LLaMA 3.3-70B) across Base, RAG- Enhanced, and Agentic methods and for each neurological subspecialties for exam 1092024.
