# Supplementary material for: A multi-agent approach to neurological clinical reasoning
Source: PLOS Digit Health. 2025 Dec 4;4(12):e0001106. doi: 10.1371/journal.pdig.0001106 (PMC12677565; doi:10.1371/journal.pdig.0001106)
Supplement: S2 File — (DOCX) [file pdig.0001106.s002.docx]

S2 File.

**Three-Dimensional Complexity Classification of 305 Neurological Board Certification Questions by Factual Knowledge Depth, Clinical Concept Integration, Reasoning Complexity and Reasoning Type.**

| **Exam** | **Q#** | **Tesxt** | **Choices** | **Correct** | **Category** | **FKD** | **CCI** | **RC** | **Reasoning Type** |
| --- | --- | --- | --- | --- | --- | --- | --- | --- | --- |
| 1052024 | 1 | Which of the following is NOT a risk factor for developing multiple sclerosis? | a. HLA-DR2 b. Low vitamin D level c. Exposure to EBV d. Migration to a more northern country after age 15 | d | Neuroimmunology | 1 | 1 | 2 | Diagnostic |
| 1052024 | 2 | Which of the following diseases is X-linked? | a. Fabry disease b. Familial ALS c. Huntington disease d. Tuberous sclerosis type 1 | a | Genetic Neurology | 1 | 1 | 1 | Diagnostic |
| 1052024 | 3 | A 28-year-old woman diagnosed with relapsing multiple sclerosis presents to the ER with limitation only in right eye adduction and nystagmus in left eye abduction. What is the location of the lesion in this condition? | a. Left Medial longitudinal fasciculus b. Right Medial longitudinal fasciculus c. Left Paramedian pontine reticular formation d. Right Paramedian pontine reticular formation | b | Neuroimmunology | 2 | 2 | 2 | Diagnostic |
| 1052024 | 4 | A 48-year-old woman had spinal cord inflammation. Brain MRI showed 10 periventricular lesions, oligoclonal bands (OCB) negative. Which of the described findings increases the patient's risk of converting from CIS to clinically definite MS? | a.Patient's age b. Brain MRI findings c. Negative OCB d. Female gender | b | Neuroimmunology | 2 | 2 | 2 | Diagnostic |
| 1052024 | 5 | In the following table, each multiple sclerosis medication is paired with its side effect. Which combination is correct? Medication / Side Effect | a. Glatiramer acetate / Recurrent infections b. Ocrelizumab / Development of autoimmune diseases c. Alemtuzumab / Depression d. Fingolimod / Cardiac rhythm disturbances | d | Neuroimmunology | 1 | 1 | 1 | Therapeutic |
| 1052024 | 6 | A multiple sclerosis patient on Natalizumab treatment has a blood test positive for JCV virus with a low titer. Which additional factor increases the risk of developing Progressive Multifocal Leukoencephalopathy (PML)? | a. Severity of white matter demyelination b. Patient's age c. Duration of natalizumab treatment d. Previous use of glatiramer acetate | c | Neuroimmunology | 1 | 3 | 2 | Diagnostic |
| 1052024 | 7 | A 40-year-old woman had two episodes of right optic neuritis in the past year. Brain MRI shows an elongated lesion in the right optic nerve involving the chiasm. No evidence of additional lesions in brain or spinal cord. Anti-AQP4 and anti-MOG antibodies were not found. Does the patient meet the 2015 criteria for NMOSD? | a. Yes, patient has two clinical events b. No, NMOSD cannot be diagnosed without antibodies c. Yes, due to the elongated lesion and chiasm involvement d. No, patient lacks spatial dissemination | d | Neuroimmunology | 2 | 3 | 2 | Diagnostic |
| 1052024 | 8 | A 45-year-old woman with breast cancer history complains of "jumps" in vision when looking forward. Examination shows arrhythmic spontaneous movements in all gaze directions. Additionally, involuntary limb movements were observed. Which antibody is most likely to be found in this patient? | a. anti-GAD b. anti-Ri c. anti-Hu d. anti-Yo | b | Neuro-oncology | 2 | 3 | 2 | Diagnostic |
| 1052024 | 9 | A 25-year-old generally healthy woman, not on regular medication, is hospitalized due to behavioral changes, memory difficulties, seizures, and involuntary movements. During hospitalization, she required intubation due to autonomic instability. What is correct regarding diagnostic tests in this patient's case? | a. In most cases, imaging in young women will show ovarian teratoma b. Most cases show hyperintense findings on brain MRI c. Most cases show Extreme delta brush pattern on EEG d. Most cases have normal CSF | a | Neuroimmunology | 2 | 2 | 2 | Diagnostic |
| 1052024 | 11 | A 17-year-old girl with schizophrenia is brought to the ER due to abnormal behavior in recent days - she moves little, remains in the same position for hours, doesn't speak at all, has rigid limbs, stares, and when placed in a position remains in it. In the ER, temperature is 39Â°C, tachycardia of 150 beats/minute, CPK normal. What is the appropriate treatment? | a. Dantrolene b. Benzodiazepines c. Discontinuation of Olanzapine d. Flumazenil | b | Behavioral & Cognitive Neurology | 1 | 2 | 2 | Therapeutic |
| 1052024 | 12 | A patient with extrapyramidal symptoms is referred for F-Dopa PET CT. The scan shows decreased uptake in the left putamen tail compared to the right. This finding may support all the following diagnoses, EXCEPT: | a. Progressive Supranuclear Palsy b. Drug induced parkinsonism c. Multiple System Atrophy d. Corticobasal degeneration | b | Movement Disorders | 2 | 2 | 2 | Diagnostic |
| 1052024 | 13 | In sporadic Creutzfeldt-Jakob disease, what is the basis for the difference between normal prion protein (PrPc) and pathological prion protein (PrPsc)? | a. Replacement of one amino acid with another b. Increased repetition of base triplets c. Change in the three-dimensional structure of the protein d. Change in mRNA level | c | Genetic Neurology | 1 | 1 | 1 | Diagnostic |
| 1052024 | 14 | A 59-year-old woman comes to the clinic for evaluation of left shoulder pain and difficulty walking over the past year. She also reports constipation and fell from bed during sleep about ten years ago. She also reports vivid dreams. Her examination shows rigidity of left arm and leg, and decreased left arm swing. What is the likely diagnosis? | a. Vascular parkinsonism b. Lewy Body Dementia c. Idiopathic Parkinson's Disease d. Multiple system atrophy | c | Movement Disorders | 2 | 3 | 2 | Diagnostic |
| 1052024 | 15 | What is the most common initial motor presentation in idiopathic Parkinson's disease? | a. Rest tremor in one hand b. Gait instability c. Slow walking d. Hand stiffness | a | Movement Disorders | 1 | 1 | 1 | Diagnostic |
| 1052024 | 16 | A 60-year-old man presents with cognitive decline accompanied by right hand tremor that began about a year ago and has worsened since. During examination, he reports visual hallucinations and daytime drowsiness. All of the following are clinical features that would support the diagnosis, EXCEPT: | a. Syncope episodes b. Eye movement disorder c. Auditory hallucinations d. Anosmia | a,b | Behavioral & Cognitive Neurology | 2 | 2 | 2 | Diagnostic |
| 1052024 | 17 | A 58-year-old woman comes for evaluation due to progressive cognitive decline in recent years. Her husband notes a history of migraines since childhood and stroke events in recent years. Her mother died from complications of a similar disease. Which of the following findings is likely to be found on brain MRI? | a. Bilateral temporal atrophy b. Gross cerebellar calcifications c. Diffusion restriction in basal ganglia d. Microbleeds | d | Genetic Neurology | 2 | 3 | 3 | Diagnostic |
| 1052024 | 18 | Which pathological protein is found in the brain of a patient suffering from chronic traumatic encephalopathy? | a. Beta-amyloid b. Tau c. TDP-43 d. Alpha-Synuclein | b | Behavioral & Cognitive Neurology | 1 | 1 | 1 | Diagnostic |
| 1052024 | 19 | A 56-year-old woman reports discomfort in her legs while lying in bed, needing to move them. The sensation is relieved by getting up and walking. Which of the following medications cannot alleviate the symptoms? | a. Opiates b. Gabapentin c. Magnesium d. Levo-dopa | c | Movement Disorders | 1 | 2 | 2 | Therapeutic |
| 1052024 | 20 | A 34-year-old woman presents with numbness in all four extremities, weakness and gait instability, confusion, hallucinations and blurred vision. Examination shows mental slowness, visual acuity disturbance, leg weakness, absent reflexes in lower extremities, bilateral extensor plantar response, impaired deep sensation in legs. Which of the following might be causing her condition? | a. Vitamin B1 deficiency b. Vitamin B3 deficiency c. Vitamin B6 deficiency d. Vitamin B12 deficiency | d | Behavioral & Cognitive Neurology | 2 | 3 | 2 | Diagnostic |
| 1052024 | 21 | In a patient with muscle weakness due to vitamin D deficiency, which findings would we expect to find in diagnostic tests? | a. Myopathic pattern on EMG b. High CPK levels in blood (>1000) c. Elevated calcium levels in blood (>10) d. Hypertrophy of muscle fibers in biopsy | a | Neuromuscular | 1 | 1 | 2 | Diagnostic |
| 1052024 | 22 | Which EMG finding is characteristic of Tick Paralysis? | a. Slow sensory conduction velocity b. Normal motor conduction velocity c. Repetitive stimulation shows decrement d. Acute denervation | b | Neuromuscular | 1 | 1 | 1 | Diagnostic |
| 1052024 | 23 | A 32-year-old man used one of the substances listed below for the first time in his life about an hour ago. He is brought to the ER by EMS after having a seizure. Which substance was he likely to have used? | a. Heroin b. Alcohol c. Cocaine d. Barbiturate | c | Epilepsy | 1 | 1 | 2 | Diagnostic |
| 1052024 | 24 | A 24-year-old woman presents with headaches for two months accompanied by visual disturbances and tinnitus. Examination: papilledema. Brain imaging CT+CTV without abnormal findings. CSF opening pressure 320 mmH2O, normal composition. Which of the following medications is associated with this condition? | a. Cephalosporin b. Tetracycline c. Quinolone d. Macrolide | b | CSF Circulation Disorders | 1 | 3 | 2 | Diagnostic |
| 1052024 | 25 | A 32-year-old woman presents with a single seizure. Reports several days of numbness around the mouth and in fingers, and involuntary muscle contractions. Examination: choreiform movements of the limbs. Which metabolic disorder could explain her symptoms? | a. Hyperthermia b. Hypoglycemia c. Hypocalcemia d. Hyperkalemia | c | Neuromuscular | 1 | 2 | 2 | Diagnostic |
| 1052024 | 26 | A 28-year-old man presents with complaints of gait instability and weakness that have been gradually worsening over several months. Examination shows abnormal eye movements, nystagmus, proximal limb weakness, ataxia, areflexia. Which nutritional deficiency explains the symptoms? | a. B12 b. Vitamin B1 c. Vitamin E d. Copper | b,c | Neuromuscular | 2 | 2 | 2 | Diagnostic |
| 1052024 | 27 | A 27-year-old woman at 30 weeks gestation presents with headache, nausea, and vomiting that developed over three days. Examination shows coarse horizontal nystagmus to the right and right-sided ataxia in arm and leg. Known to have vascular retinal findings and kidney cysts related to these neurological symptoms. What is the most likely diagnosis? | a. Hyperemesis Gravidarum b. Cerebellar tumor c. Neuromyelitis optica d. Cerebellar stroke | b | Neuro-oncology | 2 | 3 | 3 | Diagnostic |
| 1052024 | 28 | A 30-year-old woman with symptomatic myasthenia gravis, under treatment with Pyridostigmine and high-dose Prednisone, is planning pregnancy soon. Which immunosuppressive therapy is considered relatively safe in pregnancy and effective for her condition? | a. Azathioprine b. Cyclophosphamide c. Methotrexate d. Mycophenolate mofetil | a | Neuromuscular | 1 | 2 | 2 | Therapeutic |
| 1052024 | 29 | A 60-year-old woman with metastatic melanoma under chemotherapy and biological treatment and after brain and neck radiation develops severe speech and swallowing disorders. She has other symptoms including dry mouth and eyes. Symptoms improve during the day. Neurological examination: diminished tendon reflexes. Treatment with Pyridostigmine is not helpful. What pathological antibodies are expected in this case? | a. Anti-acetylcholine receptor b. Anti-Muscle Kinase c. Anti-ryanodine d. Anti-voltage gated calcium channels | a | Neuromuscular | 3 | 3 | 3 | Diagnostic |
| 1052024 | 32 | A 30-year-old woman with severe myasthenia gravis, treated with Prednisone and Azathioprine, presents with fever and diagnosis of urinary tract infection. Which antibiotic group is considered relatively safe in myasthenia? | a. Aminoglycosides b. Cephalosporins c. Macrolides d. Quinolones | b | Neuromuscular | 2 | 2 | 2 | Therapeutic |
| 1052024 | 33 | A 20-year-old man experiences recurring episodes of ataxia and dysarthria lasting several minutes and resolving. Neurological examination shows periorbital muscle rippling (muscle rippling) between attacks. Common trigger for these episodes is physical activity or startle. Similar symptoms are found in his mother. Which of the following treatments is NOT suitable as preventive treatment for this condition? | a. 4-aminopyridine b. Acetazolamide c. Benzodiazepines d. Carbamazepine | c | Genetic Neurology | 3 | 3 | 2 | Therapeutic |
| 1052024 | 34 | A 30-year-old man has bilateral hearing loss. Brain MRI shows findings consistent with bilateral acoustic neuroma. His father has a similar disease. No skin lesions were observed in the patient's physical examination. Which protein is affected in this disease? | a. Alpha fetoprotein b. Merlin c. Neurofibromin d. Tuberlin | b | Genetic Neurology | 2 | 2 | 2 | Diagnostic |
| 1052024 | 35 | A 60-year-old man has been suffering from ptosis, diplopia, speech and swallowing disorders, and limb weakness for several months. Also complains of dry mouth and eyes. Symptoms improve during the day. Neurological examination: reduced tendon reflexes. Treatment with Pyridostigmine is not effective. What pathological antibodies are expected in this case? | a. Anti-acetylcholine receptor b. Anti-Muscle Kinase c. Anti-ryanodine d. Anti-voltage gated calcium channels | d | Neuromuscular | 1 | 3 | 3 | Diagnostic |
| 1052024 | 36 | A 30-year-old woman with epilepsy is treated with Valproic acid and combined oral contraceptive. The patient is concerned that treatment with birth control pills might worsen her epilepsy. Which therapeutic approach is correct to reduce seizure risk? | a. Switch to lamotrigine treatment b. Switch to birth control pills containing estrogen above 50 micrograms c. Switch to an intrauterine device containing progesterone d. Switch to extended-release valproic acid | c | Epilepsy | 1 | 2 | 2 | Therapeutic |
| 1052024 | 37 | What is correct regarding scalp EEG recording? | a. The main normal oscillations identified are in frequencies above 40 Hz b. The source of normal rhythmic activity such as posterior alpha and spindles is from thalamic oscillators c. About 80-90 percent of cortical epileptiform activity can be identified d. The scalp mainly filters the low frequencies of EEG activity | b | Epilepsy | 1 | 2 | 2 | Diagnostic |
| 1052024 | 38 | Continuous polymorphic and non-rhythmic delta activity exclusively over the left temporal region is likely due to: | a. Normal sleep activity b. Epileptic activity c. Structural lesion in this area d. Post-anoxic state | c | Vascular Neurology | 1 | 2 | 2 | Diagnostic |
| 1052024 | 41 | Which of the following EEG findings is associated with good prognosis after anoxic damage? | a. Alpha Coma b. Burst Suppression c. Periodic Patterns d. Reactivity | d | Epilepsy | 1 | 1 | 2 | Diagnostic |
| 1052024 | 43 | Which brain location can be attributed to the symptom of Alexia with agraphia? | a. Left angular gyrus b. Left supramarginal gyrus c. Splenium of corpus callosum d. Left cingulate gyrus | a | Behavioral & Cognitive Neurology | 1 | 1 | 1 | Diagnostic |
| 1052024 | 45 | In which of the following conditions should Carotid endarterectomy be preferred over Carotid artery stenting (CAS) in cases of symptomatic extracranial carotid stenosis? | a. High carotid artery lesion b. Patients with restenosis after previous carotid surgery c. Patients over age 75 d. Patients who underwent neck radiation | c | Vascular Neurology | 1 | 2 | 2 | Therapeutic |
| 1052024 | 46 | In fundoscopic examination, what is more characteristic of pseudopapilledema and not papilledema? | a. Preserved upper disc margin b. Splinter hemorrhages c. Hyperemic color of the disc d. Abnormal vessel pattern with spontaneous venous pulsation | d | Neurophthalmology | 1 | 2 | 2 | Diagnostic |
| 1052024 | 47 | What will occur when a person with oculomotor apraxia turns their head to the right? | a. Right medial vestibular nucleus will cause inhibition in the right abducens nucleus b. Right medial vestibular nucleus will cause inhibition in the left abducens nucleus c. Left medial vestibular nucleus will cause excitation in the right abducens nucleus d. Left medial vestibular nucleus will cause excitation in the left abducens nucleus | a | Neurophthalmology | 1 | 2 | 2 | Diagnostic |
| 1052024 | 48 | A 40-year-old woman in her seventh pregnancy, at week 30, complains of neck and cervical pain and arm dysfunction that began several months before pregnancy and worsened during pregnancy. Examination: Symmetric hyperreflexia in all four limbs and bilateral Babinski sign. In forward gaze, vertical downbeat nystagmus appears. When extending arms forward with eyes closed, involuntary finger movements appear. What is the most likely diagnosis? | a. Pontine demyelination b. Foramen magnum meningioma c. Vitamin B1 deficiency d. Midbrain astrocytoma | b | Neuro-oncology | 2 | 3 | 3 | Diagnostic |
| 1052024 | 49 | In suspicion of non-organic visual field constriction, which examination method could best differentiate between non-organic tubular vision and other conditions? | a. Tangent screen b. Confrontation visual field testing c. Standard automated perimetry d. Microperimetry | a,b | Neurophthalmology | 1 | 2 | 2 | Diagnostic |
| 1052024 | 50 | A patient hospitalized after right hemispheric stroke lies continuously with closed eyes despite being conscious, follows commands and answers questions in complete sentences. When asked to open his eyes, they remain closed, but contraction of the forehead muscle with bilateral eyebrow raising is noticed. This condition is an expression of: | a. Central ptosis b. Blepharospasm c. Apraxia of eyelid opening d. Conversion reaction | c | Behavioral & Cognitive Neurology | 2 | 2 | 2 | Diagnostic |
| 1052024 | 51 | What causes Comitant exotropia (in contrast to Incomitant)? | a. Cranial nerve palsy due to diabetes b. Ocular myasthenia c. Mitochondrial disease d. Congenital strabismus | d | Neurophthalmology | 1 | 1 | 1 | Diagnostic |
| 1052024 | 52 | Which of the following findings is most likely to be caused by a lesion involving the abducens nucleus in the brainstem? | a. Facial muscle paralysis on the same side b. Increased tendon reflexes on the same side c. Horizontal nystagmus to the opposite side d. Ataxia on the same side | a | Neurophthalmology | 2 | 2 | 2 | Diagnostic |
| 1052024 | 53 | Which of the following is correct regarding Visual Evoked Potentials (VEP) testing in suspected optic nerve injury? | a. High specificity for demyelinating disease compared to other causes b. High sensitivity even for mild injuries, but low specificity c. Will be pathological only when there is significant decrease in visual acuity d. Has diagnostic value mainly in acute disease flare-up | b | Neurophthalmology | 1 | 2 | 2 | Diagnostic |
| 1052024 | 54 | A 24-year-old woman has been experiencing recurring episodes of falling in startle situations. She can only get up after several dozen seconds. Consciousness is preserved but she cannot respond. She denies excessive sleepiness. Which of the following medications might improve her condition? | a. Venlafaxine b. Nortriptyline c. Armodafinil d. Bupropion | a,b | Neuromuscular | 1 | 3 | 2 | Therapeutic |
| 1052024 | 55 | A 30-year-old man was unconscious due to CO poisoning. Examination after about 6 weeks: Breathing spontaneously, eyes open intermittently, has sleep-wake cycles. Complete lack of sphincter control. Opens eyes in response to external stimuli, can perform random limb movements that are inconsistent in response to stimuli. Does not follow commands and does not respond to questions in any way. How can this patient's state of consciousness be described? | a. Abulia b. De-efferented state c. Minimally conscious state d. Persistent vegetative state | d | Behavioral & Cognitive Neurology | 2 | 3 | 2 | Diagnostic |
| 1052024 | 57 | A 54-year-old man is in deep coma after head trauma. The question of brain death is raised. Which clinical finding rules out brain death? | a. Bilateral Babinski sign b. Pinpoint pupils c. Brisk tendon reflexes d. Triple flexion response in legs | b | Behavioral & Cognitive Neurology | 2 | 2 | 2 | Diagnostic |
| 1052024 | 58 | A 72-year-old man with Parkinson's disease complains of excessive daytime sleepiness. He is treated with Levodopa. He has symptoms of REM sleep behavior disorder (RBD), restless legs syndrome (RLS), and his sleep is disrupted due to frequent awakenings (insomnia). Which of these findings in the description is NOT related to causing excessive daytime sleepiness in the patient? | a. Levodopa b. Insomnia c. RBD-REM sleep behavior disorder d. RLS â€“ restless legs syndrome | c | Movement Disorders | 1 | 3 | 2 | Diagnostic |
| 1052024 | 59 | An 18-year-old suffers from recurring episodes that last 2-4 weeks where he sleeps many hours, and wakes only to eat and use the bathroom. When awake â€“ he is confused, eats much more than usual, and suffers from memory and behavioral disturbances. His nighttime sleep study is normal. What treatment might help prevent the episodes? | a. Bupropion b. Lithium c. Methylphenidate d. Modafinil | b | Behavioral & Cognitive Neurology | 1 | 3 | 2 | Therapeutic |
| 1052024 | 60 | What is the most common sleep disorder in hereditary and acquired muscle diseases? | a. Muscle cramps b. Sleep apnea c. Insomnia d. Restless legs syndrome | b | Neuromuscular | 1 | 1 | 1 | Diagnostic |
| 1052024 | 61 | An 83-year-old man exhibits abnormal and sometimes violent behavior during the second half of the night, at least 3 times per week. He has caused unintentional injuries to his spouse. In the past month, he fell from his bed twice during these episodes. What is the first-line medication treatment for this disorder? | a. Gabapentin b. Imipramine c. Melatonin d. Pramipexole | c | Behavioral & Cognitive Neurology | 1 | 3 | 2 | Therapeutic |
| 1052024 | 62 | A 65-year-old woman has had progressive gait difficulty for about 10 years, without orthostatic hypotension, sleep disorders, cognitive decline, or sphincter problems. No family history. Neurological examination: dysarthric speech, bilateral horizontal nystagmus, mild limb dysmetria bilaterally, brisk reflexes throughout with right Babinski sign. Normal sensation. Walks with broad base without assistive device. Extensive workup, including metabolic testing, whole-body CT, LP were normal. Brain MRI shows mild cerebellar atrophy. What is the likely diagnosis? | a. Multisystem Atrophy b. Idiopathic Late Onset Cerebellar Ataxia c. Cerebrotendinous Xanthomatosis d. Niemann Pick | b | Movement Disorders | 2 | 3 | 2 | Diagnostic |
| 1052024 | 63 | Which of the following genetic mutations that contribute to Parkinson's disease development is transmitted in an autosomal dominant manner? | a. Parkin(PARK2) b. PINK1(PARK6) c. DJ-1 (PARKK7) d. LRRK2 (PARK8) | d | Movement Disorders | 1 | 1 | 1 | Diagnostic |
| 1052024 | 64 | A 20-year-old otherwise healthy man presents with gait disturbance manifesting as inversion of the right foot during walking or running. The disturbance disappears when walking backward. What is the most likely diagnosis? | a. Dystonia b. Orthostatic tremor c. Functional movement disorder d. Tic disorder | a | Movement Disorders | 2 | 2 | 2 | Diagnostic |
| 1052024 | 65 | A 20-year-old otherwise healthy man without regular medication reports recurring episodes of rapid "body jumps" occurring during the transition between wakefulness and sleep. No similar episodes during the day. No sleep disorder. Neurological examination normal. What is the likely etiology of his complaint? | a. Physiological myoclonus b. Juvenile myoclonic epilepsy c. Essential myoclonus d. Action myoclonus | a | Movement Disorders | 1 | 1 | 1 | Diagnostic |
| 1052024 | 66 | A patient presents with partial weakness in finger extensors and triceps on the same arm, accompanied by pain and numbness of the thumb and back of hand. What is the location of the injury? | a. Radial nerve b. Radiculopathy C7 c. Posterior cord brachial plexus d. Posterior interosseus nerve | a | Neuromuscular | 2 | 2 | 2 | Diagnostic |
| 1052024 | 67 | A 45-year-old otherwise healthy man is brought to the ER after a car accident. Neurological examination â€“ conscious, cranial nerves intact, arm weakness 2/5, leg weakness 4/5, biceps and triceps reflexes not elicited, bilateral Babinski sign, cape-like sensory disturbance over upper back. What is the likely injury location according to the case description? Level / Spinal cord lesion | a. C5 / Central b. C6 / Posterior c. C5 / Anterior d. C6 / Anterior | a | Miscellaneous | 2 | 2 | 2 | Diagnostic |
| 1052024 | 70 | A soldier, age 19, healthy and strong, profile 97, complains of exercise intolerance that appears about half an hour after starting exercise. Can perform short strength training. No complaints of ptosis, diplopia, or bulbar symptoms. Examination shows no muscle weakness, atrophy, or fasciculations. Which of the following would likely show metabolic disturbance? | a. Carnitine b. Glycogen c. Fatty acid d. Mitochondria | c | Neuromuscular | 2 | 3 | 2 | Diagnostic |
| 1052024 | 71 | A 60-year-old man suffers from headaches that occur exclusively at night and wake him from sleep. The pain is bilateral, lasts an hour, without autonomic symptoms. What is the likely diagnosis? | a. Cluster headache b. Migraine c. Hypnic headache d. Primary stabbing headache | c | Headache and Dizziness | 2 | 2 | 2 | Diagnostic |
| 1052024 | 72 | A 47-year-old healthy woman reports painful contractions in calf and foot muscles, sometimes accompanied by foot deformity, usually occurring during sports classes and lasting seconds. Neurological examination is normal. NCS and EMG were performed and were normal. At the end of the examination, a calf contraction occurred, and EMG was simultaneously recorded from the gastrocnemius muscle. What is expected to be found in the recording? | a. Rapid repetitive motor unit action potential activity b. Myotonic discharge c. Positive sharp waves and fibrillation d. Electromyography silence | a | Neuromuscular | 2 | 3 | 2 | Diagnostic |
| 1052024 | 74 | A 52-year-old woman with breast cancer history has been complaining of left shoulder pain with radiation to the arm for about a month. She gradually noticed difficulty raising her left arm. Examination showed atrophy and weakness of approximately 4/5 in deltoid, biceps, triceps, supraspinatus, infraspinatus muscles on the left. No tendon reflex could be elicited from biceps and triceps on the left. EMG showed fasciculations and myokymia in recordings from these muscles. What is the most likely diagnosis? | a. Malignant infiltration of brachial plexus b. Radiation plexitis c. C5 radiculopathy d. Suprascapular nerve entrapment | a,b | Neuromuscular | 2 | 3 | 3 | Diagnostic |
| 1052024 | 75 | A 52-year-old man with chronic pain is treated with multiple medications. He developed renal colic. Which of the following medications might cause this complication? | a. Topiramate b. Lamotrigine c. Pregabalin d. Amitriptyline | a | Miscellaneous | 2 | 1 | 1 | Diagnostic |
| 1052024 | 78 | A 78-year-old man is hospitalized due to right-sided weakness and speech disorder. Examination: Alert, understands, but speaks short sentences with great effort. Has difficulty naming, writing, and repeating sentences. What type of aphasia does he have? | a. Broca aphasia b. Transcortical motor aphasia c. Conduction aphasia d. Left thalamic aphasia | a | Behavioral & Cognitive Neurology | 2 | 3 | 2 | Diagnostic |
| 1052024 | 79 | A stroke patient was asked to name a "knife" and said "fork". What is the appropriate term to describe this? | a. Semantic paraphasia b. Anomia c. Neologism d. Speech apraxia | a | Behavioral & Cognitive Neurology | 1 | 1 | 1 | Diagnostic |
| 1052024 | 80 | A 55-year-old man of Bukharian descent complains of swallowing disorder and ptosis for 5 years, difficulty rising from sitting and raising arms. No changes throughout the day. Symptoms have slowly worsened over the years. Examination shows proximal weakness in arms and legs, facial muscle weakness. What is the most likely diagnosis? | a. Oculopharyngeal muscular dystrophy b. Inclusion body myositis c. Myotonic dystrophy type II d. Lambert-Eaton syndrome | a | Neuromuscular | 2 | 3 | 2 | Diagnostic |
| 1052024 | 81 | An 18-year-old with a neurological disease that developed over several years manifests as mental disability, mild ptosis, limitation in eye movements, and gait ataxia. Retinal examination shows retinal pigmentary retinopathy. CSF protein elevated to 110 mg/dL. Which test is required as part of the workup for his condition? | a. ECG b. Blood glucose c. Urine amino acids test d. Creatinine | a | Genetic Neurology | 2 | 3 | 2 | Diagnostic |
| 1052024 | 82 | What do Ventral corticospinal tracts control? | a. Axial muscles of body posture b. External anal sphincter muscle c. Ipsilateral limb muscles d. Head movement | a | Miscellaneous | 1 | 1 | 1 | Diagnostic |
| 1052024 | 83 | Which of the following signs is NOT a sign of bladder fullness in paraplegia? | a. Excessive sweating b. Decreased pulse c. Sensation of warmth d. Increased spasticity | b,d | Neuromuscular | 1 | 2 | 2 | Diagnostic |
| 1052024 | 84 | A 60-year-old woman has been treated with steroids for many years due to lupus (SLE). Which of the following conditions can be attributed to steroid treatment? | a. Numbness in legs b. Resting tremor c. Kidney failure d. Epileptic seizures | b | Movement Disorders | 1 | 1 | 1 | Diagnostic |
| 1052024 | 85 | A 23-year-old woman comes to the ER due to gait and balance difficulties. She has a history of abdominal pain, diarrhea, and vomiting for many years. Examination shows nystagmus in all gaze directions, bilateral finger-nose test abnormality, spasticity and brisk reflexes in legs, ataxic gait. NCV testing showed axonal polyneuropathy. Which antibody would support the diagnosis? | a. Parietal cell b. Gliadin c. Glutamic acid decarboxylase d. Myelin associated glycoprotein | b | Neuroimmunology | 2 | 3 | 3 | Diagnostic |
| 1052024 | 86 | A 28-year-old man after severe head trauma deteriorates and sinks into coma within several days, without focal neurological signs. Which of the following MRI sequences has the highest diagnostic value for confirming a differential diagnosis of diffuse axonal injury? | a. Apparent diffusion coefficient (ADC) b. T1 weighted image (T1) c. Diffuse tensor imaging (DTI) d. Gradient echo (GRE) | c | Miscellaneous | 2 | 2 | 2 | Diagnostic |
| 1052024 | 87 | A 20-year-old man is admitted after spinal cord injury due to falling from height. Which combination of findings would support the diagnosis of neurogenic shock? Pulse / Tendon reflexes / Best response to treatment | a. Tachycardia / Decreased / Vasopressor b. Bradycardia / Normal / Volume replacement c. Bradycardia / Normal / Volume replacement d. Bradycardia / Decreased / Vasopressor | d | Vascular Neurology | 1 | 2 | 2 | Diagnostic |
| 1052024 | 88 | A 20-year-old man with epilepsy since early age. His physical examination showed light-colored patches on the skin and reddish, rough rash around the nose and cheeks. Which tumor is characteristic in this disease? | a. Pilocystic Astrocytoma b. Pleomorphic Xanthoastrocytoma c. Subependymal Giant Cell Astrocytoma d. Choroid Glioma of the third ventricle | c | Genetic Neurology | 2 | 2 | 2 | Diagnostic |
| 1052024 | 89 | A 68-year-old man received chemotherapy and radiation series for malignant brain glioma. After 6-8 months, his condition deteriorated, with memory deficits, gait disorder, and urinary problems. Brain MRI showed extensive white matter changes and ventricular enlargement that weren't present in previous MRI at tumor diagnosis. He improved with steroid treatment. What is the most likely diagnosis? | a. Post radiation encephalopathy b. Progressive multifocal encephalopathy c. Toxic encephalopathy d. Cytotoxic edema | a | Neuro-oncology | 1 | 3 | 3 | Diagnostic |
| 1052024 | 90 | Which of the following does NOT differentiate between Pilocytic Astrocytoma and other malignant brain tumors? | a. Degree of malignancy b. Advanced age c. Good prognosis d. Posterior fossa prevalence | b | Neuro-oncology | 1 | 2 | 2 | Diagnostic |
| 1052024 | 91 | A 48-year-old woman underwent surgery, radiation, and chemotherapy for brainstem tumor. After a year, she developed weakness in right arm and leg, with left-sided sensory disturbance and a sensory level at C5. CT PET was interpreted as normal. What is the most likely cause? | a. Post radiation myelopathy b. Chemotherapy induced myelopathy c. Paraneoplastic myelopathy d. Tumor related compressive myelopathy | a | Neuro-oncology | 2 | 3 | 3 | Diagnostic |
| 1052024 | 92 | What is the most common side effect in treatment with Temozolomide? | a. Neutropenia b. Thrombocytopenia c. Leukocytosis d. Anemia | b | Neuro-oncology | 1 | 1 | 1 | Therapeutic |
| 1052024 | 94 | Which of the following tests has the highest SENSITIVITY and SPECIFICITY for diagnosing SMALL FIBER NEUROPATHY? | a. Quantitative Sudomotor Axon reflex b. Quantitative Sensory Test of cold pain thresholds c. Electrochemical skin conductance d. Sensory nerve conduction study | a | Neuromuscular | 1 | 2 | 2 | Diagnostic |
| 1052024 | 95 | A 45-year-old man was admitted due to severe headache followed by loss of consciousness. Brain CT showed subarachnoid hemorrhage. Physical examination showed small hemorrhages in the nail bed and a new systolic murmur. At admission: temperature 39Â°C and 20,000 leukocytes in blood. What is the most likely pathogen causing this clinical picture? | a. ASPERGILLUS b. CANDIDA c. VIRIDANS STREP d. STREP PNEUMONIA | c | Infectious Neurology | 2 | 3 | 2 | Diagnostic |
| 1052024 | 97 | A 30-year-old HIV-positive man complains of cognitive decline. HIV infection was diagnosed at intermediate stage (CD4 cell count of 200-500/microliter). Direct brain involvement of which of the following viruses is most likely to explain his symptoms at this stage of his disease? | a. CMV b. HIV c. JC-virus d. VZV | b | Behavioral & Cognitive Neurology | 2 | 2 | 2 | Diagnostic |
| 1052024 | 98 | A 40-year-old AIDS patient with CD4 count of 35 cells/microliter presents with lower back pain radiating to legs and urinary retention. Examination shows bilateral foot drop and absent Achilles reflexes. What is the recommended treatment? | a. Acyclovir b. Famicyclovir c. Oseltamivir d. Ganciclovir | d | Infectious Neurology | 1 | 2 | 2 | Therapeutic |
| 1052024 | 99 | A 45-year-old man with history of intravenous drug use is admitted due to high fever, severe headache and confusion that developed over the past month. CSF shows 300 mononuclear cells, protein 150 mg/dL, glucose 37 mg/dL (blood glucose 100 mg/dL). Brain MRI shows diffuse enhancement of meninges, especially in basal cisterns. What is the most likely pathogen? | a. Cryptococcus neoformans b. HIV c. Toxoplasma gondii d. Mucormycosis | a | Infectious Neurology | 2 | 3 | 2 | Diagnostic |
| 1052024 | 100 | A 58-year-old patient on anticoagulation due to mechanical valve presents with large right lobar hemorrhage. INR is 4.8. Which of the following poses a risk for further expansion of the cerebral hemorrhage? | a. Amount of edema around the hemorrhage b. Location of hemorrhage c. Enhancement of artery in hemorrhage area d. Evidence of multiple additional small hemorrhages | c | Vascular Neurology | 2 | 3 | 2 | Diagnostic |
| 1092024 | 1 | Which genetic disease is caused by a repetition of four nucleotides? | a. Myotonic dystrophy type 2 b. Huntington disease c. Spinocerebellar ataxia type 1 d. Friedreich ataxia | a | Genetic Neurology | 1 | 1 | 1 | Diagnostic |
| 1092024 | 2 | Which of the following genetic diseases is not inherited through mitochondrial inheritance? | a. Duchenne muscular dystrophy b. Kearns-Sayre syndrome c. LHON (Leber hereditary optic neuropathy) d. MERRF (myoclonic epilepsy with ragged red fibers) | a | Genetic Neurology | 1 | 1 | 1 | Diagnostic |
| 1092024 | 3 | A 25-year-old woman was diagnosed with optic neuritis in the right eye. Brain MRI shows three lesions around the ventricles (periventricular) and a lesion at the C2 level without enhancement. What would help diagnose the patient with relapsing multiple sclerosis according to the 2017 McDonald criteria? | a. The patient meets the criterion for the diagnosis of relapsing multiple sclerosis. b. Positive result for oligoclonal bands in cerebrospinal fluid. c. Hyperintense lesion of the right optic nerve on MRI. d. Prolonged P100 in visual evoked potentials of the right optic nerve. | b | Neuroimmunology | 2 | 2 | 3 | Diagnostic |
| 1092024 | 4 | Which of the following is not a prognostic factor for a more severe course of multiple sclerosis? | a. Family history. b. Patient's gender. c. Type of clinical attack. d. Number of attacks. | a | Neuroimmunology | 1 | 2 | 2 | Diagnostic |
| 1092024 | 5 | In the following table, each multiple sclerosis drug is listed with its mechanism of action. Which combination is incorrect? | a. Fingolimod, mechanism of action: S1P receptor modulator b. Ocrelizumab, mechanism of action: anti-CD20 c. Alemtuzumab, mechanism of action: anti-CD52 d. Dimethyl fumarate, mechanism of action: inhibition of pyrimidine synthesis | d | Neuroimmunology | 1 | 1 | 1 | Diagnostic |
| 1092024 | 6 | A 17-year-old male with no prior medical history, one week after receiving an influenza vaccine, presents with symptoms of fever, confusion, seizures, right-sided hemiparesis, and ataxia. Lumbar puncture reveals 50 white blood cells, mostly lymphocytes, with no evidence of infectious etiology in ancillary tests. What is most likely to be seen on brain MRI? | a. Hypointense lesions on T1 sequence b. Extensive leptomeningeal enhancement c. Confluent lesions with enhancement d. Small diffuse hemorrhagic foci in the posterior fossa | c | Neuroimmunology | 2 | 3 | 3 | Diagnostic |
| 1092024 | 7 | A 51-year-old man complains of stiffness and muscle spasms. Examination reveals significant axial rigidity that worsens with touch or loud noise. Electrophysiological testing shows continuous motor unit activity in the involved muscles. Which antibody is most commonly found in this syndrome? | a. Anti-amphiphysin b. Anti-CRMP5 c. Anti-GAD d. Anti-glycine receptor | c | Neuroimmunology | 2 | 3 | 2 | Diagnostic |
| 1092024 | 8 | A 60-year-old smoker with no regular medication presents with progressive leg weakness and difficulty climbing stairs over several weeks, along with dry mouth and erectile dysfunction. Examination reveals proximal limb weakness, more pronounced in the legs than arms, and reduced reflexes. Which antibody is expected to be found? | a. Anti-titin b. Anti-MuSK c. Anti-AChR d. Anti-VGCC | d | Neuromuscular | 2 | 3 | 3 | Diagnostic |
| 1092024 | 9 | Which multiple sclerosis drug works by disrupting lymphocyte entry into the central nervous system? | a. Glatiramer acetate b. Dimethyl fumarate c. Interferon beta 1A d. Natalizumab | d | Neuroimmunology | 1 | 1 | 1 | Therapeutic |
| 1092024 | 10 | A 58-year-old under neurological follow-up complains of uncontrollable episodes of laughing and crying, sometimes without context. All of the following neurological diseases could explain his condition, except: | a. Multiple sclerosis b. Motor neuron disease c. Multiple brain infarcts', image_path=None), Choice(label='d', text="Huntington's disease", image_path=None)] | d | Behavioral & Cognitive Neurology | 2 | 2 | 2 | Diagnostic |
| 1092024 | 11 | A 41-year-old man suspects that security agents are eavesdropping on his conversations, hears voices commanding him to stay home, and believes news broadcasts are sending him personal messages. Symptoms have persisted for two years, and neurological evaluation was normal. Which of the following is true regarding the epidemiology of the disease he is suffering from? | a. Drug use increases the risk of developing the disease. b. Living in a rural area increases the risk of developing the disease. c. Being born in summer increases the risk of developing the disease. d. His twin brother has about an 80% risk of developing the same disease. | a | Behavioral & Cognitive Neurology | 2 | 2 | 2 | Diagnostic |
| 1092024 | 12 | A patient with epilepsy and hypertension presents to the clinic reporting low mood, loss of appetite, anhedonia, and insomnia. Among the following medications, which is the most appropriate treatment option for him? | a. Escitalopram b. Venlafaxine c. Bupropion d. Mirtazapine | d | Epilepsy | 1 | 2 | 3 | Therapeutic |
| 1092024 | 13 | Among the diagnostic tests supporting the diagnosis of Creutzfeldt-Jakob disease, which test has the highest specificity? | a. MRI b. Total tau protein in CSF c. 14-3-3 protein in CSF d. RT-QuIC | d | Behavioral & Cognitive Neurology | 1 | 2 | 1 | Diagnostic |
| 1092024 | 14 | Which of the following genes causes early-onset Alzheimer's disease inherited in an autosomal dominant manner? | a. APP b. APOE c. TARDBP d. ABCA7 | a | Behavioral & Cognitive Neurology | 1 | 1 | 1 | Diagnostic |
| 1092024 | 15 | Which of the following findings at the onset of the disease would lead to a probable diagnosis of Parkinson-plus syndrome as opposed to idiopathic Parkinson's disease? | a. Hypophonia b. Asymmetry of findings on examination c. Frontal release signs d. Significant gait instability | d | Movement Disorders | 1 | 2 | 2 | Diagnostic |
| 1092024 | 16 | An 18-year-old reports frequent blinking, throat clearing, and nose pulling that appear intermittently and fluctuate over the years. The patient is at increased risk for the following disorders, except: | a. Tendency for compulsiveness and obsessive thoughts b. Sensitivity to background noise and difficulty maintaining concentration c. Impulsivity and tendency for substance use d. Somatoform and conversion disorders | d | Movement Disorders | 2 | 2 | 2 | Diagnostic |
| 1092024 | 17 | Which of the following medications is used as a first-line treatment for chorea? | a. VMAT2 inhibitors b. D2-receptor blockers c. Benzodiazepines d. Baclofen | a,b | Movement Disorders | 1 | 1 | 1 | Therapeutic |
| 1092024 | 18 | A 75-year-old woman is evaluated in the clinic for cognitive decline with short-term memory impairment. On examination, when asked to pantomime how to brush her teeth, she demonstrates how to comb her hair. What is the most likely diagnosis? | a. Frontotemporal Dementia b. Alzheimer's Disease c. Diffuse Lewy Body Disease d. Right Parietal Stroke | b | Behavioral & Cognitive Neurology | 2 | 2 | 2 | Diagnostic |
| 1092024 | 19 | A 75-year-old woman is evaluated in the clinic. Motor examination is normal. When asked to demonstrate how to make a cup of coffee, she is unable to do so. What is the disorder? | a. Ideational apraxia b. Limb kinetic apraxia c. Conceptual apraxia d. Motor optic apraxia | a,c | Behavioral & Cognitive Neurology | 2 | 3 | 2 | Diagnostic |
| 1092024 | 20 | A 48-year-old man with a history of hypertension is found unconscious on the street. Upon EMS arrival, cardiac arrest is noted on the monitor. He undergoes resuscitation. Which of the following signs, present after 24 hours, does not indicate a very poor prognosis? | a. Weakness in the upper limbs b. Dilated pupils c. Generalized myoclonus d. Upward gaze deviation | a | Vascular Neurology | 2 | 3 | 2 | Diagnostic |
| 1092024 | 21 | A 62-year-old man with liver cirrhosis due to hepatitis C is admitted due to new-onset confusion that worsened in the days prior to hospitalization. Laboratory tests show impaired liver function and elevated ammonia levels. What characterizes ammonia metabolism in this context? | a. Blood ammonia level does not correlate with the severity of encephalopathy b. Normal ammonia levels rule out hepatic encephalopathy c. Blood pH level affects the brain's ability to clear ammonia d. Ammonia detoxification occurs mainly in the kidneys | a,c | Behavioral & Cognitive Neurology | 2 | 2 | 2 | Diagnostic |
| 1092024 | 22 | A 33-year-old woman, 17 weeks pregnant, presents with severe vomiting, progressively worsening confusion over several days, and unsteady gait. On examination, there is impairment of eye movements, horizontal and vertical nystagmus, and a wide-based gait. What finding is expected on brain MRI? | a. Acute infarction in the pons b. Filling defects in bilateral transverse sinuses c. Demyelinating lesion in the middle cerebellar peduncle d. Hyperintense finding on FLAIR sequence in the periaqueductal region | d | Behavioral & Cognitive Neurology | 2 | 3 | 2 | Diagnostic |
| 1092024 | 23 | A 45-year-old man who works in a battery manufacturing plant presents due to gradual development over several weeks of bilateral wrist drop. He denies any sensory disturbance or pain. On physical examination, which of the following findings might be discovered? | a. A pale line in the fingernails b. A blue line in the gums c. Polycythemia d. Pyramidal signs | b | Neuromuscular | 2 | 2 | 2 | Diagnostic |
| 1092024 | 24 | A 64-year-old woman with a history of opioid use due to malignancy. Which of the following signs support opioid withdrawal syndrome as opposed to opioid intoxication? | a. Nausea and vomiting b. Miosis c. Sweating d. Tachypnea | d | Behavioral & Cognitive Neurology | 1 | 1 | 2 | Diagnostic |
| 1092024 | 25 | Which of the following does not cause cytotoxic cerebral edema? | a. Trauma b. Liver failure c. Stroke d. Brain malignancy | d | Vascular Neurology | 1 | 2 | 1 | Diagnostic |
| 1092024 | 26 | A 64-year-old man with a history of hypertension is hospitalized due to a cerebellar stroke presenting with gait instability. Treated with tPA. Two days after admission, he develops headache, decreased consciousness, and complaints of diplopia. On examination, blood pressure is 200/110 mmHg, pulse is 35 bpm, and he is drowsy. An urgent brain CT is performed. Which finding explains his condition? | a. Ventricular enlargement b. Extensive frontal infarction c. Subdural hemorrhage d. Bilateral occipital edema | a | CSF Circulation Disorders | 2 | 3 | 3 | Diagnostic |
| 1092024 | 27 | A 25-year-old woman with muscle weakness and positive antibodies against MuSK. Partial improvement in symptoms was observed after treatment with prednisone. Which of the following treatments is expected to significantly improve her condition? | a. Eculizumab b. Intravenous Immunoglobulin c. Pyridostigmine d. Rituximab | b,d | Neuromuscular | 1 | 2 | 2 | Therapeutic |
| 1092024 | 28 | A 40-year-old woman with a history of migraines and drug use is hospitalized due to subcortical hemispheric hemorrhage. A CTA study demonstrated 'beading' of intracranial arteries. Use of which of the following drugs is most commonly associated with the described clinical picture? | a. Cocaine b. Opioids c. Cannabinoids d. Amphetamine | a,d | Vascular Neurology | 2 | 2 | 2 | Diagnostic |
| 1092024 | 29 | An 18-year-old man has experienced symptoms since the age of 2, including ptosis, diplopia, speech difficulties, swallowing difficulties, and limb weakness worsening in the evening. Blood tests for acetylcholine receptor antibodies were negative. Steroid treatment and plasma exchange were ineffective. Improvement was observed after treatment with pyridostigmine and amifampridine. Which pathological finding is expected in this case? | a. Positive antibodies to MuSK b. Positive antibodies to voltage-gated calcium channels c. Mutation in the acetylcholine receptor gene d. Mutation in the acetylcholine esterase enzyme gene | c | Neuromuscular | 3 | 3 | 3 | Diagnostic |
| 1092024 | 30 | A 30-year-old woman with suspected functional (non-organic) weakness on the right side. Which neurological examination finding supports the suspected diagnosis? | a. In Hoover's test, lifting the left leg causes extension of the right leg. b. Stable gait with difficulty standing on toes and heels. c. Weakness in the leg is more pronounced in flexor muscles compared to extensor muscles. d. In the arm position test, pronation of the right hand is observed without dropping. | a | Behavioral & Cognitive Neurology | 2 | 2 | 2 | Diagnostic |
| 1092024 | 32 | A 20-year-old man with recurrent episodes of significant limb weakness lasting about an hour and resolving. Neurological examination reveals difficulty opening the fist after strong and prolonged muscle contraction. In a muscle exercise test, the CMAP amplitude on EMG increases with each effort. However, in a prolonged exercise test, there is a temporary increase in amplitude followed by a prolonged decrease lasting 40 minutes. What is the diagnosis? | a. Hyperkalemic periodic paralysis b. Hypokalemic periodic paralysis c. Myotonia congenita d. Paramyotonic congenita | a | Neuromuscular | 2 | 3 | 3 | Diagnostic |
| 1092024 | 34 | A 20-year-old male with a large nevus in the area of the forehead and right eye, cognitive decline, and epileptic seizures since childhood. Which of the following tumors is associated with the described disease? | a. Acoustic Neuroma b. Hemangioblastoma c. Leptomeningeal angioma d. Optic Glioma | c | Genetic Neurology | 2 | 2 | 2 | Diagnostic |
| 1092024 | 36 | A 20-year-old female suffering from migraines with aura, non-smoker. The patient is interested in treatment with oral combined contraceptives. In which case of aura can this treatment be given without concern for an increased risk of stroke? | a. Aura with main manifestations of diplopia and vertigo. b. Aura with visual disturbances including flickering, flashes, and glare. c. Aura with manifestations of visual disturbances, aphasia, and memory impairment. d. Aura with manifestations of weakness in the arm and leg. | b | Headache and Dizziness | 1 | 2 | 2 | Therapeutic |
| 1092024 | 37 | What does EEG activity originate from? | a. The sum of intracellular inhibitory and excitatory currents. b. The sum of extracellular currents at inhibitory and excitatory synapses. c. The sum of action potentials traveling along axons. d. The sum of action potentials on the neuronal cell bodies. | b | Miscellaneous | 1 | 1 | 1 | Diagnostic |
| 1092024 | 38 | Which of the following reduces posterior alpha activity? | a. Eye closure. b. Chronic treatment with barbiturates. c. Drowsiness. d. Darkness. | c | Behavioral & Cognitive Neurology | 1 | 2 | 2 | Diagnostic |
| 1092024 | 43 | A 23-year-old man has been suffering from drug-resistant epilepsy for 4 years. In childhood, he experienced febrile seizures. Video EEG demonstrates seizures during which he presses the alert button due to an epigastric sensation, followed by right head turning, left hand dystonia, oral automatisms, and right hand automatisms. The seizure lasts about a minute. After the seizure, he is confused but answers questions. What is the most likely source of his seizures? | a. Right mesial temporal. b. Left mesial temporal. c. Left temporal neocortex. d. Right temporal neocortex. | a | Epilepsy | 2 | 3 | 2 | Diagnostic |
| 1092024 | 45 | A 14-year-old girl has been experiencing seizures in the left side of her body for a year, with progressive weakness in the left side and atrophy of the right hemisphere. Antibodies against which receptor can be found in the described case? | a. Glutamate. b. GABA. c. Dopamine. d. Serotonin. | a | Neuroimmunology | 3 | 3 | 3 | Diagnostic |
| 1092024 | 46 | A 60-year-old man presented to the emergency department with fever, severe cough, chest pain, and headache. Neurological examination revealed a right pupil smaller than the left, both reactive to light, and mild right ptosis. Which of the following findings supports the etiology of carotid artery dissection compared to a lung apex tumor? | a. Lack of dilation of the right pupil after instillation of cocaine drops. b. Lack of constriction of the right pupil after instillation of pilocarpine drops. c. Lack of dilation of the right pupil after instillation of hydroxyamphetamine drops. d. Lack of constriction of the right pupil after instillation of apraclonidine drops. | c | Neurophthalmology | 2 | 2 | 2 | Diagnostic |
| 1092024 | 47 | In which of the following diseases is the sense of smell usually preserved? | a. Idiopathic Parkinson disease. b. Multiple system atrophy (MSA) type P. c. Vascular parkinsonism. d. Huntington disease. | c,d | Movement Disorders | 1 | 2 | 2 | Diagnostic |
| 1092024 | 48 | A 42-year-old aircraft mechanic complains of hearing loss, which he attributes to exposure to engine noise. What pattern in the hearing test is more typical and specific for this type of damage? | a. Selective loss at low frequencies. b. Gradual down-sloping loss at high frequencies. c. Notch around 4000 Hz. d. Uniform loss across all frequency ranges. | c | Headache and Dizziness | 1 | 1 | 1 | Diagnostic |
| 1092024 | 49 | A 56-year-old male with hypertension and diabetes presents with a sudden sharp decrease in vision in the left eye, without pain. On fundoscopic examination of the left eye, the optic disc appears edematous and whitish with hemorrhages between the fibers. The right eye's optic disc is normal. Laboratory results: HbA1C = 7.8%, ESR and CRP are normal. What is the expected finding in the visual field test for this patient? | a. Enlargement of the blind spot in the left eye. b. Concentric narrowing of the visual field in the left eye. c. Deficit in the upper visual field of the left eye. d. Central scotoma in the left eye and deficit in the upper temporal field of the right eye. | c | Neurophthalmology | 2 | 3 | 2 | Diagnostic |
| 1092024 | 50 | A 40-year-old female complains of pain in the right orbit for several weeks and double vision. On examination: mild proptosis of the right eye, mild periorbital edema. CT of the orbits shows thickening of the extraocular muscles without any other significant findings. What is the most common finding in eye movement examination in this condition? | a. Limitation in adduction of the eyeball due to involvement of the medial rectus. b. Limitation in abduction of the eyeball due to involvement of the medial rectus. c. Limitation in elevation of the eyeball due to involvement of the superior oblique. d. Limitation in depression of the eyeball due to involvement of the superior oblique. | a,b,c,d | Neurophthalmology | 2 | 3 | 2 | Diagnostic |
| 1092024 | 51 | What is the role of the orbitofrontal cortex in the sense of taste? | a. Differentiation between sweet and salty tastes. b. Integration of information from taste pathways with other sensory information. c. Processing specific information originating from the fungiform papillae on the tongue. d. Serving as the primary taste cortex. | b | Behavioral & Cognitive Neurology | 1 | 2 | 2 | Diagnostic |
| 1092024 | 52 | What is the expected finding in the acoustic reflex test in the case of mild to moderate cochlear damage on the right? | a. Absence of motor response of the reflex to auditory stimulation on the right. b. Motor response of the reflex only to auditory stimulation at a higher intensity than normal. c. Absence of motor response of the reflex to any auditory stimulation. d. Motor response of the reflex to auditory stimulation at a lower intensity than normal. | b,d | Neurophthalmology | 1 | 2 | 2 | Diagnostic |
| 1092024 | 53 | A 72-year-old male with a history of hypertension and heavy smoking presents to the emergency department with sudden onset of dizziness, nausea, vomiting, sharp hearing loss in the left ear, oscillopsia, facial numbness on the left, and coordination disturbance. On examination: weakness of the facial muscles and decreased sensation on the left side of the face, deafness in the left ear, nystagmus, ataxia on the finger-to-nose test on the left, and decreased pinprick sensation in the right arm and leg. Which artery is affected? | a. Left posterior inferior cerebellar artery (PICA). b. Left superior cerebellar artery (SCA). c. Left anterior inferior cerebellar artery (AICA). d. Median branches of the left basilar artery. | c | Vascular Neurology | 2 | 3 | 3 | Diagnostic |
| 1092024 | 54 | A 73-year-old male with vascular risk factors complains of multiple falls without preceding symptoms or loss of consciousness. He feels his legs collapse, 'sees the ground approaching,' and finds himself on the floor. Additionally, he reports tremors in the right limbs after mild exertion. CTA of the cervical vessels shows 80%-99% stenosis in the left internal carotid artery (LICA). What additional finding explains the described falls and limb tremors? | a. Dominant right vertebral artery and narrow left vertebral artery. b. Origin of both anterior cerebral arteries from a common A1 segment on the left. c. Stenosis in the A2 segment of the left anterior cerebral artery. d. Hypoplasia of the right posterior communicating artery. | b | Vascular Neurology | 3 | 3 | 3 | Diagnostic |
| 1092024 | 55 | A 72-year-old male hospitalized after a myocardial infarction begins to exhibit disorganized thinking and incoherent speech on the second day of hospitalization. He experiences visual hallucinations, hyperarousal at night, and psychomotor suppression during the day. He is disoriented to time and place and does not know why he is in the hospital. Which cognitive domain is most significantly impaired in the syndrome he is experiencing? | a. Attention. b. Memory. c. Perception. d. Planning. | a | Behavioral & Cognitive Neurology | 1 | 2 | 2 | Diagnostic |
| 1092024 | 56 | A 64-year-old male was admitted in a state of unconsciousness. On eye examination, round, equal pupils of intermediate size (neither dilated nor constricted) were observed, which did not respond to light. At what level is the structural lesion causing these findings? | a. Thalamus b. Midbrain c. Pons d. Medulla | b | Neurophthalmology | 2 | 2 | 2 | Diagnostic |
| 1092024 | 57 | A 30-year-old male is in a state of unconsciousness following severe head trauma. Which drug has the potential to improve functional outcomes in patients in his condition? | a. Amphetamine b. Amantadine c. Apomorphine d. Amitriptyline | b | Behavioral & Cognitive Neurology | 2 | 2 | 1 | Therapeutic |
| 1092024 | 58 | Which type of memory impairment characterizes an episode of transient global amnesia? | a. Impairment of immediate memory (working or immediate memory) b. Impairment of short-term memory (recent memory) c. Impairment of long-term memory (remote memory) d. Impairment of procedural memory (motor or procedural memory) | b | Behavioral & Cognitive Neurology | 1 | 1 | 1 | Diagnostic |
| 1092024 | 59 | A 19-year-old female, generally healthy, complains of recurrent episodes several times a day of muscle weakness lasting 1â€“2 minutes. The weakness can cause falls, jaw dropping, head dropping, or speech disturbance. The episodes usually occur during emotionally charged events (laughter, excitement, anger). She is not sleepy during the day. What is expected to be found on EEG at the beginning of the episode? | a. Background slowing and appearance of sleep spindles b. Focal ictal epileptic activity c. Normal wakefulness activity d. Generalized ictal epileptic activity | c | Neuromuscular | 2 | 3 | 2 | Diagnostic |
| 1092024 | 60 | A 58-year-old male with restless legs syndrome also suffers from depression. What would be the antidepressant of choice (in the absence of contraindications)? | a. Amitriptyline b. Bupropion c. Fluoxetine d. Venlafaxine | b | Movement Disorders | 1 | 2 | 2 | Therapeutic |
| 1092024 | 61 | In the evaluation of a patient with ataxia, which imaging finding would suggest a diagnosis of Fragile X Tremor Ataxia Syndrome? | a. T2-hyperintensity in the bilateral middle cerebellar peduncles b. T2-hyperintensity in the mammillary bodies, periaqueductal gray, and paraventricular thalamus c. T2-hyperintensity in the bilateral inferior olivary nucleus d. T2-hyperintensity in the corticospinal tract | a | Genetic Neurology | 2 | 2 | 2 | Diagnostic |
| 1092024 | 62 | An 18-year-old male experiences recurrent involuntary movements in the neck and limbs, presenting as dystonia or dyskinesia. On some days, there are dozens of similar episodes, usually triggered by standing up from sitting or changing position. Each episode lasts seconds to a few minutes, without loss of consciousness and with full memory of the event. He denies an urge to perform involuntary movements. EEG was normal, even during a typical episode. What is the recommended treatment? | a. Carbamazepine b. Ketogenic diet c. Haloperidol d. Clonidine | a | Movement Disorders | 1 | 3 | 2 | Therapeutic |
| 1092024 | 63 | Which of the following types of tremor is particularly characteristic of idiopathic Parkinson's disease? | a. Kinetic tremor b. Re-emergent tremor c. Jerky postural tremor d. Titubation | b | Movement Disorders | 1 | 1 | 1 | Diagnostic |
| 1092024 | 66 | A 40-year-old woman, generally healthy, complains of weakness in her right hand for several months. Recently, weakness in her left leg also appeared. On examination: weakness and atrophy with fasciculations, no sensory disturbance, no autonomic involvement, and no sphincter involvement. No bulbar involvement. No pyramidal signs. NCS/EMG showed demyelinating changes with conduction blocks in areas that are non-compression sites. Lumbar puncture revealed no cells, protein 70. How should the investigation proceed? | a. Anti-MAG antibodies test b. Anti-GM1 antibodies test c. Anti-GD1b antibodies test d. Anti-GQ1b antibodies test | b | Neuromuscular | 2 | 3 | 2 | Diagnostic |
| 1092024 | 67 | A 20-year-old man was examined in the emergency department after being involved in a car accident with a dorsal injury. On examination: no weakness in the hands, right leg weakness 4/5 with Babinski sign on the right, reduced pain sensation in the left leg, reduced vibration and position sense in the right leg. Based on the symptoms, which part of the spinal cord is affected and at what level? | a. C3 left b. T3 left c. C3 right d. T3 right | d | Miscellaneous | 2 | 3 | 2 | Diagnostic |
| 1092024 | 68 | A patient presents with bilateral leg weakness and lower back pain. What would indicate conus medullaris syndrome compared to cauda equina syndrome? | a. Saddle anesthesia b. Incontinence c. Pyramidal signs d. Muscle tone | c | Neuromuscular | 2 | 2 | 2 | Diagnostic |
| 1092024 | 69 | Which muscle groups are more likely to be involved in inclusion body myositis? | a. Finger flexors and knee extensors b. Knee flexors and elbow extensors c. Ankle dorsiflexors and wrist extensors d. Hip flexors and finger extensors | a | Neuromuscular | 1 | 1 | 2 | Diagnostic |
| 1092024 | 70 | A 42-year-old healthy man, engaged in sports, consulted a physiotherapist due to right buttock pain radiating to the back of the leg. Suspected piriformis syndrome was raised. Which diagnostic manipulation is likely to reproduce the pain? | a. Low back extension b. Straight leg raising c. Flexion and adduction of the hip d. Hip extension | c | Neuromuscular | 1 | 1 | 2 | Diagnostic |
| 1092024 | 71 | A 40-year-old woman suffers from short episodes of pain occurring in different parts of the head, mainly above the eye. Each episode lasts seconds and resolves. The patient also suffers from migraines. What is the recommended preventive treatment? | a. Indomethacin b. Beta blocker c. Carbamazepine d. Verapamil | a | Headache and Dizziness | 1 | 3 | 2 | Therapeutic |
| 1092024 | 72 | A 65-year-old man is hospitalized in intensive care, intubated and sedated due to pneumonia. Laboratory monitoring revealed CPK levels around 15,000 with myoglobinuria. Which of the following drugs is the most likely causal factor? | a. Midazolam b. Propofol c. Fentanyl d. Norepinephrine | b | Neuromuscular | 2 | 2 | 2 | Diagnostic |
| 1092024 | 73 | A 47-year-old man complains of neck pain radiating to the arms for about a month. On examination: decreased pain and temperature sensation in the hands, normal position sense, and the rest of the examination is normal. What is the most likely diagnosis? | a. HTLV1 infection b. Ependymoma c. Copper deficiency d. Spinal meningioma | b | Neuro-oncology | 2 | 2 | 2 | Diagnostic |
| 1092024 | 74 | What is the main neurotransmitter of nociceptive C-fibers? | a. Glutamate b. Norepinephrine c. Substance P d. GABA | c | Neuromuscular | 1 | 1 | 1 | Diagnostic |
| 1092024 | 75 | What is the mechanism of action of baclofen in the treatment of trigeminal neuralgia? | a. NMDA antagonist b. GABA-b agonist c. NMDA agonist d. GABA-a antagonist | b | Neuromuscular | 1 | 1 | 1 | Therapeutic |
| 1092024 | 76 | A 65-year-old woman has been suffering from headaches, weight loss, and low-grade fever for a month and a half. She was admitted to the emergency department due to sudden vision loss in the right eye with narrowing of the upper visual field. Elevated ESR was noted. What is the most likely diagnosis? | a. Susac syndrome b. APLA syndrome c. Giant cell arteritis d. Neuro-sarcoidosis | c | Vascular Neurology | 2 | 3 | 2 | Diagnostic |
| 1092024 | 78 | A 62-year-old woman is evaluated in the memory clinic due to memory disturbances and comprehension difficulties progressing over two years. Her speech fluency is relatively preserved, but she struggles to name and understand even single words. FDG PET imaging shows bilateral temporal hypometabolism, more prominent on the left. What is the diagnosis? | a. Semantic dementia b. Progressive non-fluent aphasia c. Logopenic progressive aphasia d. Pure word deafness | a | Behavioral & Cognitive Neurology | 2 | 3 | 2 | Diagnostic |
| 1092024 | 79 | Which bedside test can be performed to assess speech apraxia? | a. Ask the patient to repeat the word 'television' five times. b. Ask the patient to lick their lips in a circular motion. c. Ask the patient to say a sentence and then write it down. d. Ask the patient to drink a glass of water and then speak. | a | Behavioral & Cognitive Neurology | 1 | 1 | 1 | Diagnostic |
| 1092024 | 80 | Which neurotransmitter is primarily involved in mediating excitotoxicity? | a. Glutamate b. Noradrenaline c. Dopamine d. Acetylcholine | a | Miscellaneous | 1 | 1 | 1 | Diagnostic |
| 1092024 | 81 | What characterizes brain infarcts in mitochondrial diseases? | a. Cortical infarcts b. Watershed distribution infarcts c. Venous infarcts d. Infarcts in the territory of large arteries | a | Genetic Neurology | 3 | 2 | 1 | Diagnostic |
| 1092024 | 82 | A 66-year-old woman has had hand weakness for a year and recently developed walking difficulties. On examination: dysarthria, muscle atrophy in all four limbs, fasciculations in the muscles, and increased tone in the legs. Which of the following is NOT consistent with her symptoms? | a. Bulbospinal neuropathy (Kennedy disease) b. ALS - Amyotrophic lateral sclerosis c. Adult hexosaminidase-A deficiency d. Adult polyglucosan body disease | a | Neuromuscular | 3 | 3 | 3 | Diagnostic |
| 1092024 | 83 | A 35-year-old man sustained a spinal cord injury and remains with paraplegia and difficulty urinating. All the following actions can help him urinate except: | a. Tapping on the bladder b. Rubbing the skin in the pubic area and inner thighs c. Applying pressure to the abdominal wall d. Performing the Valsalva maneuver | a,b,c,d | Miscellaneous | 1 | 1 | 1 | Therapeutic |
| 1092024 | 84 | What is the most common neurological complication of rheumatoid arthritis? | a. Mononeuropathy multiplex b. Compression neuropathies c. Distal sensorimotor polyneuropathy d. Small fiber neuropathies | a,b,c | Neuromuscular | 1 | 1 | 2 | Diagnostic |
| 1092024 | 86 | A 50-year-old male presents to the ER after being involved in a fight and stabbed in the lower right side of his back very close to the spine. MRI shows signs of hemorrhage in the right side of the spinal cord at the level of D10 on the right. | a. Left leg weakness and pyramidal signs, decreased pain and temperature sensation on the right below the stab level, decreased position sense on the left below the stab level b. Right leg weakness and pyramidal signs, decreased pain and temperature sensation on the right below the stab level, decreased position sense on the left below the stab level c. Right leg weakness and pyramidal signs, decreased pain and temperature sensation on the left below the stab level, decreased position sense on the right below the stab level d. Right leg weakness and pyramidal signs, decreased pain and temperature sensation on the right below the stab level, decreased position sense on the right below the stab level | c | Vascular Neurology | 2 | 2 | 2 | Diagnostic |
| 1092024 | 88 | A 20-year-old female with known kidney cysts and retinal changes, diagnosed with kidney failure at a young age. Which tumor is common in this disease? | a. Medulloblastoma b. Pilocytic astrocytomas c. Hemangioblastoma d. Choroid plexus tumors | c | Genetic Neurology | 2 | 2 | 2 | Diagnostic |
| 1092024 | 89 | A 30-year-old male complains of progressive leg weakness and sensory disturbances. Examination shows paraparesis. Sensory examination reveals sacral sparing. Imaging shows a tumor near the conus medullaris. What is the most likely primary tumor? | a. Ependymoma b. Schwannoma c. Meningioma d. Glioma | a | Neuro-oncology | 2 | 3 | 2 | Diagnostic |
| 1092024 | 90 | What is the most effective initial treatment for Primary Central Nervous System Lymphoma in patients without AIDS? | a. methotrexate b. cyclophosphamide c. rituximab d. methylprednisolone | a | Neuro-oncology | 1 | 1 | 2 | Therapeutic |
| 1092024 | 91 | A 64-year-old male with metastatic lung cancer began complaining of hoarseness, swallowing difficulty, and weakness of the trapezius and sternocleidomastoid muscles. Where is the metastasis most likely to be seen on brain MRI? | a. Occipital condyle b. Jugular foramen c. Hypoglossal foramen d. Foramen magnum | b | Neuro-oncology | 2 | 2 | 2 | Diagnostic |
| 1092024 | 93 | A 30-year-old male with right lower facial muscle weakness. EMG examination of cranial nerve VII showed neurogenic changes in the affected muscles. Where is the most likely pathological location? | a. Left cortex b. Right pons c. Right cerebellopontine angle d. Right parotid | d | Neuromuscular | 2 | 2 | 2 | Diagnostic |
| 1092024 | 94 | A 20-year-old male fell asleep on his arm on a park bench after using drugs. He woke up with right wrist drop. The weakness resolved one month later. What was the location of nerve injury? | a. Myelin b. Vaso nervosum c. Perineurium d. Axon | a | Neuromuscular | 2 | 3 | 2 | Diagnostic |
| 1092024 | 95 | A 70-year-old man presented to the emergency department with complaints of double vision and eyelid drooping. On examination: complete ptosis of the left eye, outward deviation of the left eyeball, and dilation of the left pupil. An aneurysm in which of the following arteries would explain his condition? | a. Superior cerebellar artery b. Vertebral artery c. Ophthalmic artery d. Anterior communicating artery | a | Neurophthalmology | 2 | 2 | 2 | Diagnostic |
| 1092024 | 96 | A 27-year-old woman, generally healthy, one month postpartum. Recently, due to low mood, she started treatment with an SSRI. She was admitted due to a severe headache that began suddenly with transient motor aphasia. On admission: her neurological examination was normal, non-contrast brain CT was normal, and CSF composition showed protein 35 mg/dL and 3 lymphocytic cells. What is the most likely diagnosis? | a. Transient headache with neurological deficits and CSF lymphocytosis b. Reversible cerebral vasoconstriction syndrome c. Mitochondrial encephalomyopathy with lactic acidosis and stroke-like episodes d. Cerebral sinus vein thrombosis | b | Vascular Neurology | 2 | 3 | 2 | Diagnostic |
| 1092024 | 97 | A 35-year-old man with AIDS and a very low CD4 count started treatment with cART. One week after starting treatment, he experienced significant cognitive deterioration. What is the most likely explanation? | a. Direct neurotoxic effect of cART b. Development of progressive multifocal leukoencephalopathy (PML) c. Vasculitis secondary to HIV d. Immune reconstitution inflammatory syndrome (IRIS) | d | Infectious Neurology | 1 | 2 | 3 | Diagnostic |
| 1092024 | 98 | What is the most common post-infectious autoimmune syndrome associated with COVID-19? | a. Acute necrotizing encephalopathy (ANE) b. Acute disseminated encephalomyelitis (ADEM) c. Guillain-BarrÃ© syndrome (GBS) d. Acute myelitis | c | Neuroimmunology | 1 | 1 | 1 | Diagnostic |
| 1092024 | 99 | A 56-year-old man with poorly controlled diabetes presented to the emergency department with severe worsening back pain over the past week. On examination: localized tenderness over the thoracic spine, without focal neurological deficits. ESR was 70 mm/hour. MRI showed a rounded mass in the epidural space with peripheral pathological enhancement, adjacent to the spinal cord, with signs of discitis. What is the most common causative organism? | a. Streptococcus pneumoniae b. Mycobacterium tuberculosis c. Escherichia coli d. Staphylococcus aureus | d | Infectious Neurology | 2 | 3 | 2 | Diagnostic |
| 1092024 | 100 | For which of the following patients is decompressive craniectomy recommended as part of stroke treatment? | a. A 90-year-old man with acute pontine hemorrhage and decreased consciousness. b. A 70-year-old man with acute cerebellar hemorrhage due to an arteriovenous malformation, fully conscious. c. A 55-year-old man with left middle cerebral artery occlusion and decreased consciousness. d. A 70-year-old woman with acute basal ganglia hemorrhage and decreased consciousness. | c | Vascular Neurology | 1 | 2 | 2 | Therapeutic |
| 1062023 | 1 | A 70-year-old man with hand tremor that started 10 years ago. The tremor is mainly action-related. No rigidity or slowness. Which of the following is against the diagnosis of essential tremor? | a. Asymmetric tremor b. Similar tremor in the father c. Tremor during writing d. Tremor in the lower limbs | d | Movement Disorders | 2 | 3 | 2 | Diagnostic |
| 1062023 | 3 | A 42-year-old man working in a metal factory presents with fatigue, drowsiness, tremor, generalized slowness, and falls. On examination, parkinsonian syndrome is observed. Poisoning with which of the following substances could cause his condition? | a. Lead b. Arsenic c. Manganese d. Thallium | c | Movement Disorders | 2 | 2 | 2 | Diagnostic |
| 1062023 | 4 | A 63-year-old man is evaluated for memory decline. Rapid plasma reagin and fluorescent treponemal antibody absorption tests are positive. Neurological examination is normal, and he denies a history of sexually transmitted diseases. Which diagnostic test is appropriate in this case? | a. Serology for antiphospholipid antibodies (APLA) b. Brain MRI c. Lumbar puncture d. EEG | c | Infectious Neurology | 2 | 3 | 2 | Diagnostic |
| 1062023 | 5 | A 30-year-old woman complains of headaches for the past month. On examination: papilledema is observed, with no abnormal findings on brain imaging. Which of the following factors could be associated with her symptoms? | a. Use of the antibiotic Amoxicillin b. Use of oral contraceptives c. Use of Eltroxin d. Use of vitamin D | b | CSF Circulation Disorders | 2 | 2 | 2 | Diagnostic |
| 1062023 | 6 | Which of the following findings is characteristic in muscle biopsy of patients with critical myopathy illness? | a. Necrosis of type 1 fibers b. Loss of thick myosin filament c. Absence of vacuolation d. Perivascular inflammation | b | Neuromuscular | 1 | 1 | 1 | Diagnostic |
| 1062023 | 7 | Which of the following tumors typically appears before the age of 20? | a. Glioblastoma b. Optic nerve glioma c. Hemangiopericytoma d. Vestibular schwannoma | b | Neuro-oncology | 1 | 1 | 1 | Diagnostic |
| 1062023 | 8 | A 55-year-old woman with no medical history complains of memory disturbances. Her mother has Alzheimerâ€™s disease, which started at age 77. The patient reports forgetting where she placed her keys and forgetting names. She functions well as a schoolteacher and at home. On MOCA testing, her final score is 29/30. What is her risk of developing Alzheimerâ€™s disease? | a. No increased risk b. Slightly increased compared to others her age c. 10% to 20% per year d. 50% by age 70 | b | Behavioral & Cognitive Neurology | 1 | 1 | 2 | Diagnostic |
| 1062023 | 9 | The parents of a 17-year-old boy report that recently he has been sleeping most of the day and waking only to eat. They describe him eating large amounts of food and then returning to sleep. They note a similar episode 8 months ago. Which of the following medications has been found effective as preventive treatment for this condition? | a. Valproic acid b. Lithium c. Melatonin d. Paroxetine | b | Behavioral & Cognitive Neurology | 1 | 3 | 2 | Therapeutic |
| 1062023 | 10 | A 30-year-old man with a psychiatric illness taking multiple medications presents for neurological consultation due to prolonged neck dystonia. Which treatment should be discontinued? | a. Lithium b. Phenothiazine c. Valproic Acid d. Escitalopram | b | Movement Disorders | 1 | 2 | 2 | Therapeutic |
| 1062023 | 11 | A 60-year-old man has experienced gradual functional decline over the past year, characterized by difficulty recognizing people, reading, writing, and depth perception. What additional finding is expected in the neurological examination? | a. Difficulty recognizing colors b. Delusional thoughts c. Decline in semantic memory d. Inappropriate social behavior | a | Behavioral & Cognitive Neurology | 2 | 2 | 2 | Diagnostic |
| 1062023 | 12 | A 15-year-old girl who did not receive routine childhood vaccinations develops a febrile illness with a sore throat. Examination of the throat reveals a rigid grayish exudate. A week later, she develops nasal speech, difficulty swallowing, and blurred vision. Which ophthalmologic finding is most likely in this patient? | a. Nystagmus b. Argyll Robertson pupil c. Loss of accommodation d. Sixth nerve paralysis | c | Neuromuscular | 2 | 3 | 3 | Diagnostic |
| 1062023 | 13 | A 65-year-old woman with rheumatoid arthritis treated with methotrexate and prednisone has experienced recurrent cerebral infarctions in recent months. Brain MRI shows infarcts of varying ages in different vascular territories, primarily at the gray-white matter junction. Which infection is likely responsible for the vasculopathy in this patient? | a. Cytomegalovirus b. Herpes simplex virus c. Human herpesvirus 6 d. Varicella-zoster virus | d | Infectious Neurology | 2 | 2 | 3 | Diagnostic |
| 1062023 | 14 | A 26-year-old man presents with a two-week history of headaches located at the back of the head and neck, worsened by standing and relieved by lying down. Neurological examination is unremarkable. Lumbar puncture shows an opening pressure of 40 mm H2O with normal CSF content. Which MRI finding supports the suspected diagnosis? | a. Enhancement in the basal ganglia b. Bilateral subarachnoid hemorrhage c. Subdural effusion in the temporal lobes d. Generalized ventricular enlargement | c | CSF Circulation Disorders | 2 | 3 | 2 | Diagnostic |
| 1062023 | 15 | A 63-year-old man presents with leg muscle pain accompanied by decreased sensation in the feet. He has been on long-term medication. Neurological examination reveals proximal leg weakness, reduced pain sensation in a stocking distribution, and absent Achilles reflexes. Which of the following medications can the patient continue taking without worsening his neurological condition? | a. Colchicine b. Propranolol c. Amiodarone d. Chloroquine | b | Neuromuscular | 1 | 3 | 2 | Therapeutic |
| 1062023 | 16 | The combination of deletions in chromosomes 1p and 19q is associated with: | a. Worse response to chemotherapy in oligodendroglioma tumors b. Worse response to chemotherapy in anaplastic astrocytoma tumors c. Better response to chemotherapy in anaplastic astrocytoma tumors d. Better response to chemotherapy in oligodendroglioma tumors | d | Neuro-oncology | 1 | 1 | 1 | Diagnostic |
| 1062023 | 17 | A 30-year-old male was involved in a motor vehicle accident with a head injury. A CT scan at the time of the accident revealed a linear occipital fracture. Three months later, he complains of headaches and severe memory impairment. Neurological examination shows that the patient cannot recall family members' names, birth dates, ID numbers, or names of animals. In a word memory test, he recalls 5/5 words after 5 minutes. What is the explanation for the patient's symptoms? | a. Diffuse axonal injury b. Chronic subdural hemorrhage c. Hydrocephalus d. Malingering | d | Behavioral & Cognitive Neurology | 2 | 3 | 2 | Diagnostic |
| 1062023 | 18 | A 40-year-old male with epilepsy complains of chronic pain over the past few months. Which treatment can be added without concern for worsening his epilepsy? | a. Imipenem b. Tramadol c. Bupropion d. Paroxetine | d | Epilepsy | 1 | 2 | 2 | Therapeutic |
| 1062023 | 19 | In which of the following conditions is conduction block not observed in nerve conduction studies? | a. Ulnar compression neuropathy b. Chronic inflammatory demyelinating polyneuropathy c. Multifocal motor neuropathy d. Charcot-Marie-Tooth disease | d | Neuromuscular | 1 | 2 | 2 | Diagnostic |
| 1062023 | 20 | A patient with light-colored hair and mild intellectual disability presents with sudden onset of monocular blurred vision. Ophthalmologic examination reveals lens subluxation. What is the most likely diagnosis? | a. Mucopolysaccharidosis b. Myotonic dystrophy c. Galactosemia d. Homocystinuria | d | Genetic Neurology | 2 | 2 | 2 | Diagnostic |
| 1062023 | 21 | A 35-year-old male presents with hyperkinetic movement disorders and behavioral changes. Examination reveals generalized chorea involving the limbs, trunk, and face. His father had a similar condition that began at age 55. Which genetic mutation is associated with this disease? | a. Multiple CAG repeats on the short arm of chromosome 4 b. Multiple CGG repeats on the X chromosome c. Multiple CAG repeats on the short arm of chromosome 12 d. Multiple CTG repeats on the short arm of chromosome 19 | a | Genetic Neurology | 2 | 2 | 2 | Diagnostic |
| 1062023 | 22 | A 43-year-old man working in agriculture presented to the hospital with complaints of abdominal pain, vomiting, excessive salivation, sweating, and headache. On examination: miosis and diffuse weakness. What neurological complication might develop later? | a. Cognitive decline b. Cerebellar syndrome c. Parkinsonism d. Corticospinal damage | c,d | Neuromuscular | 2 | 2 | 2 | Diagnostic |
| 1062023 | 24 | A 55-year-old man with diffuse muscle disease developed rigidity, high fever, and rapid pulse after general anesthesia. What is the diagnosis? | a. Central core myopathy b. Nemaline rod myopathy c. Centronuclear myopathy d. Myofibrillar myopathy | a | Neuromuscular | 2 | 2 | 2 | Diagnostic |
| 1062023 | 25 | A 45-year-old man complains of headaches for three months. A CT scan shows a right frontal space-occupying lesion surrounded by a hypodense area in the white matter, which appears hyperintense on T2-weighted MRI and with reduced anisotropy on DWI. What can be found in this area? | a. Swelling of neurons and glial cells b. Leakage of plasma proteins from capillaries into the extracellular space c. Decrease in extracellular fluid volume d. Higher permeability to albumin compared to low molecular weight substances | b | Neuro-oncology | 2 | 2 | 2 | Diagnostic |
| 1062023 | 26 | Which of the following axonal neuropathies of metabolic/toxic origin is characterized by predominantly proximal motor involvement? | a. Thiamine deficiency b. Vitamin B12 deficiency c. Porphyria d. Chemotherapy-induced neuropathy | c | Neuromuscular | 1 | 2 | 2 | Diagnostic |
| 1062023 | 27 | A 70-year-old man describes increasing difficulty initiating walking, with a sensation that his feet are 'sticking to the ground.' Additionally, he is very afraid of falling, and there is significant improvement in his ability to walk when another person lightly holds his hand. Neurological examination in bed is normal. What is likely to be found in auxiliary tests? | a. SPECT - reduced uptake in the temporal and parietal lobes b. Brain MRI - small vessel disease mainly in the frontal region c. Brain CT - midbrain atrophy with relatively preserved pons volume d. EMG - early recruitment of small units in proximal leg muscles | b | Movement Disorders | 2 | 3 | 2 | Diagnostic |
| 1062023 | 28 | A 45-year-old woman with urinary incontinence for over 10 years and subsequent severe spastic paraparesis. Recently, she has been experiencing mild cognitive decline. Brain MRI shows extensive symmetrical confluent changes in the white matter. What is the likely diagnosis? | a. Adult polyglucosan body disease b. Tropical spastic paraparesis c. Primary progressive multiple sclerosis d. CADASIL | a | Genetic Neurology | 2 | 3 | 3 | Diagnostic |
| 1062023 | 29 | A 52-year-old oncology patient undergoing chemotherapy complains of dizziness and unsteadiness while walking. On examination: dysarthria, nystagmus, cerebellar dysfunction in all four limbs, and gait ataxia. The symptoms resolved after the treatment ended. Which drug caused her condition? | a. 5-Fluorouracil b. Vincristine c. Procarbazine d. L-asparaginase | a | Neuro-oncology | 1 | 3 | 2 | Diagnostic |
| 1062023 | 30 | A 67-year-old woman is being treated for chronic neuropathic pain with duloxetine. In which pain transmission and control system does the drug primarily act in this case? | a. Anterior spinothalamic tract b. Descending pain-control system c. Spinoreticulothalamic tract d. Thalamic ventroposterior nuclei | b | Neuromuscular | 2 | 2 | 1 | Diagnostic |
| 1062023 | 31 | All of the following statements about the neurotransmitter acetylcholine are correct EXCEPT: | a. Secreted at all preganglionic fibers in parasympathetic ganglia b. Secreted at all preganglionic fibers in sympathetic ganglia c. Secreted at all postganglionic fibers in parasympathetic ganglia d. Secreted at all postganglionic fibers in sympathetic ganglia | d | Miscellaneous | 1 | 1 | 1 | Diagnostic |
| 1062023 | 32 | A 60-year-old man with a history of hypertension is brought to the emergency department due to rapid onset of severe weakness in all four limbs. On examination: lying with eyes open, following only vertical gaze, occasionally blinking, unable to speak, quadriplegia, and bilateral Babinski sign. Where is the lesion located? | a. Ventral pons b. Cerebral peduncles c. Medial medulla d. Cervico-medullary junction | a | Vascular Neurology | 2 | 2 | 2 | Diagnostic |
| 1062023 | 33 | What is the most common tumor in the pineal region? | a. Pineocytoma b. Atypical pineocytoma c. Germinoma d. Pinealoblastoma | c | Neuro-oncology | 1 | 1 | 1 | Diagnostic |
| 1062023 | 35 | Which of the following actions would be impaired by an isolated injury to the musculocutaneous nerve? | a. Extension of the forearm b. Flexion of the forearm in a supinated position c. Abduction of the arm up to 90 degrees d. Extension of the wrist | b | Neuromuscular | 1 | 1 | 1 | Diagnostic |
| 1062023 | 36 | Which neurotransmitter is secreted by Purkinje cells in the cerebellum? | a. Dopamine b. GABA c. Glutamate d. Acetylcholine | b | Miscellaneous | 1 | 1 | 1 | Diagnostic |
| 1062023 | 37 | A patient with a brain tumor has a visual field defect with a central scotoma on the side of the tumor and a contralateral superior temporal quadrant defect. Which area is the tumor compressing? | a. Optic tract b. Optic nerve c. Optic nerve-chiasm junction d. Optic radiation | c | Neuro-oncology | 2 | 2 | 2 | Diagnostic |
| 1062023 | 46 | A 70-year-old man has been suffering from Parkinson's disease for several years. He is treated with several medications and develops leg edema. Which of the following drugs is most likely to have caused this? | a. Entacapone b. Rasagiline c. Levodopa d. Amantadine | d | Movement Disorders | 2 | 2 | 2 | Diagnostic |
| 1062023 | 48 | Blood pressure regulation according to changes in body position is carried out by two types of baroreceptors. One is located in the aortic arch and carotid sinus, and the other is located in the right side of the heart and pulmonary vessels. Which of the following is correct regarding the baroreceptors? | a. Baroreceptors in the carotid sinus are sensitive to an increase in pressure pulse b. Baroreceptors in the pulmonary vessels and the right side of the heart respond to changes in blood volume c. Baroreceptors in the carotid sinus have a slow response time d. Baroreceptors in the aortic arch have a fast response time | b | Miscellaneous | 1 | 2 | 2 | Diagnostic |
| 1062023 | 49 | A 20-year-old woman underwent a brain MRI due to complaints of headaches. The MRI demonstrated a space-occupying lesion in the left temporo-parietal region. Which of the following findings in the tumor biopsy indicates a good prognosis? | a. A combination of astrocytes and oligodendrocytes b. Bipolar astrocytic cells, hair-like c. Positive staining for GFAP d. Absence of mutations in IDH1 and IDH2 | b | Neuro-oncology | 2 | 2 | 2 | Diagnostic |
| 1062023 | 51 | A 20-year-old man is admitted to the emergency department due to worsening limb weakness over two days. On examination: oral temperature 38.6Â°C, pulse 110 regular, blood pressure 130/80, fully conscious, mild neck stiffness, normal gross strength and tone in the arms, weakness in the right leg and left leg (4+/5), areflexia in all stations, plantar reflexes flexor, normal sensation. Brain CT is normal. Lumbar puncture: 60 cells with a predominance of lymphocytes, protein 110 mg/dL, normal glucose. Which of the following diagnoses is most likely? | a. Guillain-Barre syndrome b. Enterovirus infection c. Tick paralysis d. Mycoplasma myelitis | a | Neuromuscular | 2 | 3 | 2 | Diagnostic |
| 1062023 | 52 | A 77-year-old man with a history of smoking and hypertension has been experiencing vomiting and diarrhea in recent days. He arrives at the emergency department due to sudden onset weakness in the right side of the body, involving the shoulder and thigh more than the face. What is most likely to be found in the CTA of the neck and brain? | a. Occlusion of the left MCA b. Severe stenosis of the left ICA c. Occlusion of the left ACA d. Dissection of the left vertebral artery | c | Vascular Neurology | 2 | 2 | 2 | Diagnostic |
| 1062023 | 53 | A 68-year-old man complains of body stiffness, tremor in the right hand, and a change in handwriting (it has become smaller). Which eye movement disorder might be found in this patient? | a. Internuclear ophthalmoplegia b. Saccadic pursuit c. Supranuclear gaze paresis | b | Movement Disorders | 2 | 2 | 2 | Diagnostic |
| 1062023 | 54 | A 67-year-old man has been experiencing progressive weakness in all four limbs for six months. On examination: muscle atrophy, fasciculations, weakness in all four limbs with brisk tendon reflexes, bilateral Babinski sign, normal sensation. Brain and cervical/thoracic spine MRI are normal. What is the expected finding in NCS/EMG? | a. Conduction block b. Prolonged distal latency c. Increased recruitment d. Enlarged motor units | d | Neuromuscular | 2 | 3 | 2 | Diagnostic |
| 1062023 | 61 | A 50-year-old man is hospitalized in intensive care and suffers from multi-organ failure. During his hospitalization, he develops symmetric polyneuropathy. What characterizes this diagnosis? | a. Typical involvement of cranial nerves b. Significant autonomic involvement is rare c. The neuropathy is demyelinating d. Painful sensory involvement is common | b | Neuromuscular | 2 | 2 | 2 | Diagnostic |
| 1062023 | 62 | Which of the following is NOT a manifestation of striatocapsular infarction? | a. Contralateral hemiparesis b. Motor aphasia c. Gaze preference d. Homonymous hemianopsia | c | Vascular Neurology | 1 | 2 | 2 | Diagnostic |
| 1062023 | 63 | A 26-year-old woman complains of headaches and binocular horizontal diplopia when looking to the right, especially when viewing distant objects. Which of the following is NOT a possible cause of her complaints? | a. Posterior communicating artery aneurysm b. Post lumbar puncture c. Idiopathic intracranial hypertension d. Chronic meningitis | a | Neurophthalmology | 2 | 2 | 2 | Diagnostic |
| 1062023 | 64 | A 25-year-old man, two days after a car accident, suffers from flaccid tetraplegia and loss of sphincter control. On examination, no deep tendon reflexes are elicited in the limbs, and there is no plantar response. Which reflex is expected to return first? | a. Patellar b. Achilles c. Babinski d. Bulbocavernosus | d | Neuromuscular | 2 | 2 | 2 | Diagnostic |
| 1062023 | 65 | A 30-year-old generally healthy woman, 27 weeks pregnant, suffers from a fever up to 38Â°C, general weakness, and dizziness. After a few days, she develops gait instability, followed by diplopia. On examination: mild peripheral facial weakness on the right and limitation in abduction of the right eye. Brain CT is normal. Lumbar puncture shows lymphocytic pleocytosis with normal protein and glucose levels. Brain MRI shows a hyperintense area on T2 in the brainstem, most prominent at the medulla-pons junction. Which of the following pathogens is the cause of her condition? | a. Borrelia burgdorferi b. Listeria monocytogenes c. Neisseria meningitidis d. Treponema pallidum | b | Infectious Neurology | 2 | 3 | 3 | Diagnostic |
| 1062023 | 66 | A 68-year-old man with a history of hypertension and atrial fibrillation, untreated, is admitted due to deviation of the head and eyes to the left. Subsequently, there is extension of the left arm and leg. Where is the likely epileptic focus? | a. Left frontal b. Left temporal c. Right temporal d. Right frontal | d | Epilepsy | 2 | 2 | 2 | Diagnostic |
| 1062023 | 67 | A 78-year-old woman underwent shunt placement for suspected normal pressure hydrocephalus (NPH) three months ago. She now presents with severe headaches, vomiting, and altered consciousness. Brain imaging shows significant ventricular enlargement compared to previous imaging and chronic subdural hemorrhage without change from prior imaging. What is the most likely cause of her condition? | a. Shunt infection b. Subdural hemorrhage c. Shunt obstruction d. Vertebrobasilar infarction | c | CSF Circulation Disorders | 2 | 3 | 3 | Diagnostic |
| 1062023 | 69 | Which of the following findings would appear in radiation necrosis and help differentiate it from tumor recurrence? | a. Absence of uptake on PET scan b. Lack of enhancement with gadolinium on MRI c. Mass with high vascularization on angiography d. Increased cerebral blood volume on perfusion studies | a | Neuro-oncology | 1 | 2 | 2 | Diagnostic |
| 1062023 | 70 | A patient after a head injury with brain tissue contusion draws a clock as follows: Which pathological result in the following tests would indicate damage in the same area? | a. Color naming b. Figure Copying Test c. Speech-sound perception d. Verbal fluency | b | Behavioral & Cognitive Neurology | 2 | 2 | 2 | Diagnostic |
| 1062023 | 71 | An 18-year-old male presents with recurrent abdominal pain, behavioral changes, psychosis, and limb weakness. On examination: upper limb weakness with areflexia. Urine test shows high levels of delta-amino-levulinic acid (delta-ALA). What is the treatment for his condition? | a. IV Immunoglobulins b. Plasmapheresis c. IV Glucose + Hematin d. IV methylprednisolone | c | Genetic Neurology | 1 | 3 | 2 | Therapeutic |
| 1062023 | 73 | In an Optokinetic Nystagmus (OKN) test, nystagmus appears when the film moves from right to left but not when it moves from left to right. Where is the likely lesion located? | a. Flocculo-nodulus b. Left parietal lobe c. Right occipital lobe d. Tegmentum of pons | b | Neurophthalmology | 2 | 2 | 2 | Diagnostic |
| 1062023 | 74 | A 37-year-old male has had progressive leg weakness for two weeks, along with weight loss and cough. On examination: asymmetric spastic paraparesis. MRI of the thoracic spine shows a long lesion in the cord with multifocal sub-pial nodular enhancements near the lesion. Chest CT shows enlarged lymph nodes in the pulmonary hilum. What is the likely diagnosis? | a. Neuromyelitis Optica b. Paraneoplastic associated myelitis c. Polyangiitis with eosinophilia d. Sarcoidosis | d | Neuroimmunology | 2 | 3 | 3 | Diagnostic |
| 1062023 | 75 | A 28-year-old female recovering from encephalitis due to herpes simplex virus (HSV) infection begins to improve gradually. Suddenly, she develops confusion similar to before and drowsiness, along with worsening focal epileptic seizures. Which test might reveal the cause of the deterioration? | a. Antiâ€“myelin oligodendrocyte glycoprotein antibody b. Anti-aquaporin 4 antibody c. Anti-voltage gated calcium channel antibodies d. Antiâ€“N-methyl-D-aspartate (NMDA) receptor antibody | d | Neuroimmunology | 2 | 2 | 2 | Diagnostic |
| 1062023 | 76 | A 48-year-old male complains of recurrent episodes of visual hallucinations of shapes accompanied by intense anger. What is the most likely diagnosis? | a. Old frontal infarction b. Occipital tumor c. Temporal cavernoma d. Parietal meningioma | c | Behavioral & Cognitive Neurology | 2 | 2 | 2 | Diagnostic |
| 1062023 | 77 | A 55-year-old male presents with muscle pain that began two months after starting a new medication. Which of the following drugs could be the cause of these symptoms? | a. Citalopram b. Lithium c. Quetiapine d. Venlafaxine | b | Neuromuscular | 1 | 1 | 1 | Diagnostic |
| 1062023 | 78 | A 25-year-old man is found to have a red port-wine stain in the area of the left upper eyelid. What are the clinical features associated with this condition? | a. Reduced reflexes in the ipsilateral limbs to the stain b. Focal seizure c. Resting tremor in the limbs d. Cerebellar signs | b | Genetic Neurology | 2 | 2 | 2 | Diagnostic |
| 1062023 | 79 | Which of the following is associated with the appearance of anxiety symptoms? | a. Increased secretion of corticotropin-releasing hormone b. Decreased secretion of aldosterone c. Increased secretion of follicular stimulating hormone d. Decreased secretion of urinary epinephrine | a | Behavioral & Cognitive Neurology | 1 | 1 | 1 | Diagnostic |
| 1062023 | 80 | A 25-year-old woman in advanced pregnancy arrives at the emergency room with a severe headache. On examination: she cannot see in both eyes, there is no blink reflex when a fast-approaching object or strong light is directed at either eye. Pupillary examination shows good response to light bilaterally. Fundoscopy reveals no disc edema. There is no limitation in eye movements. What is the most likely diagnosis? | a. Neuromyelitis optica b. Factitious visual disorder c. Pituitary apoplexy d. Posterior reversible encephalopathy | d | Vascular Neurology | 2 | 3 | 3 | Diagnostic |
| 1062023 | 81 | Which of the following mononeuropathies is the most common in diabetic neuropathy? | a. Oculomotor b. Peroneal c. Radial d. Facial | a | Neuromuscular | 1 | 1 | 1 | Diagnostic |
| 1062023 | 82 | An 80-year-old woman was brought to the emergency room with altered consciousness. MRI diffusion sequence demonstrated the following finding. Occlusion of which artery could explain the described condition? | a. Huebner b. Percheron c. Pericallosal d. Lenticulostriate | b | Vascular Neurology | 2 | 2 | 2 | Diagnostic |
| 1062023 | 83 | A 30-year-old woman presents for evaluation of right-sided ptosis. Upon lifting the right eyelid, left eyelid ptosis is observed. What is the most likely diagnosis? | a. Thyroid disease b. Myasthenia gravis c. Hornerâ€™s syndrome d. Third nerve palsy | b | Neuromuscular | 2 | 2 | 2 | Diagnostic |
| 1062023 | 84 | A 45-year-old generally healthy woman suddenly experienced neck pain radiating to the arms, followed by flaccid weakness in both arms. Later, symptoms of dizziness and nausea appeared. Cervical spine MRI showed a hyperintense signal in the anterior part of the spinal cord, including the anterior horns, on T2 sequence, with restriction on DWI sequence. What is the most likely diagnosis? | a. Acute disseminated encephalomyelitis b. Spinal stroke c. Neuromyelitis optica d. Spinal hemorrhage | b | Vascular Neurology | 2 | 3 | 3 | Diagnostic |
| 1062023 | 85 | A 45-year-old man with AIDS complains of lower back pain, numbness, and later bilateral weakness in the lower limbs. Symptoms developed over two weeks following a febrile illness, without rash. Examination reveals diffuse leg weakness, reduced reflexes in the knees and ankles. Additionally, retinal inflammation was found on eye examination. Lumbar puncture shows elevated protein and mild pleocytosis, predominantly mononuclear cells. What is the most common pathogen causing this condition? | a. Cytomegalovirus b. Epstein-Barr virus c. Human immunodeficiency virus d. Varicella-zoster virus | a | Infectious Neurology | 2 | 3 | 3 | Diagnostic |
| 1062023 | 86 | A 10-year-old boy, generally healthy, appears older than his age with axillary and groin hair. He was referred to a psychiatrist due to uncontrollable laughing episodes, including during school and in inappropriate situations. What is the likely etiology? | a. Craniopharyngioma b. Olfactory Meningioma c. Hypothalamic Hamartoma d. Cholesteatoma | c | Behavioral & Cognitive Neurology | 2 | 3 | 2 | Diagnostic |
| 1062023 | 87 | A person who regularly consumes raw meat presents with generalized weakness, diffuse muscle pain, dysarthria, and double vision. On examination: facial edema, eye misalignment, mild proximal muscle weakness in the arms, dysarthria, and tongue weakness. Elevated CK levels and EMG findings show fibrillations and small polyphasic motor units. What is the causative agent of this disease? | a. Trichinella spiralis b. Toxoplasma gondii c. Taenia solium d. Schistosoma mansoni | a | Neuromuscular | 2 | 3 | 2 | Diagnostic |
| 1062023 | 88 | A 74-year-old man with gait disturbance and cognitive decline complains of uncontrollable crying and laughing episodes without a clear reason. Which of the following medications could help in this condition? | a. Dextromethorphan/Quinidine b. Levodopa/Carbidopa c. Haloperidol/Biperiden d. Fluoxetine/Clonazepam | a | Behavioral & Cognitive Neurology | 1 | 2 | 2 | Therapeutic |
| 1062023 | 90 | A 40-year-old man with chronic limb weakness. On examination: proximal and distal weakness graded 4/5, areflexia, no pathological reflexes, sensory disturbance in a glove-and-stocking distribution, and hair loss in the lower limbs. Which EMG/NCV finding would support a diagnosis of genetic neuropathy? | a. Nerve conduction block b. CMAP dispersion c. Electromyographic polyphasic waves d. Uniform slow nerve conduction velocity | d | Genetic Neurology | 2 | 2 | 2 | Diagnostic |
| 1062023 | 91 | A 75-year-old man complains of gradual hearing loss in both ears over the past year. What is the most common cause of hearing loss in this patient? | a. Loss of spiral ganglion neurons b. Atrophy of Heschlâ€™s gyrus c. Bilateral ossification of the auditory bones d. Vestibular schwannoma | a | Behavioral & Cognitive Neurology | 1 | 1 | 1 | Diagnostic |
| 1062023 | 93 | A 54-year-old female migrant worker from South America presents with numbness and tingling in the fourth and fifth fingers of her right hand. These symptoms have been present for several months and have gradually worsened. On examination: mild atrophy of the hypothenar eminence, a thickened nerve palpable in the right cubital tunnel, and several raised, oval, red lesions scattered on the skin. What is the most likely diagnosis? | a. Leprosy b. Carpal tunnel syndrome c. Ulnar nerve entrapment d. Peripheral neuropathy | c | Neuromuscular | 2 | 3 | 2 | Diagnostic |
| 1062023 | 94 | A 53-year-old woman has had tremor, slowness, and instability while walking for two years, which have gradually worsened with falls. On examination: extrapyramidal and cerebellar signs. She reports dizziness with a feeling of near-fainting when transitioning from sitting to standing. Orthostatic hypotension is noted on blood pressure measurement. Which of the following medications cannot help alleviate her orthostatic hypotension? | a. Atropine b. Droxidopa c. Midodrine d. Fludrocortisone | a | Movement Disorders | 1 | 3 | 2 | Therapeutic |
| 1062023 | 95 | A 60-year-old man presents with difficulty swallowing and progressive weakness in his limbs. On examination: weakness of the quadriceps, flexor pollicis longus, and neck extensors. CK = 300 IU. EMG shows fibrillations with small polyphasic motor units. Which of the following is not characteristic of the muscle biopsy findings in the described case? | a. Immune complexes deposited in the walls of arterioles b. Intracytoplasmic subsarcolemmal vacuoles c. Eosinophilic inclusions in cytoplasm and nuclei d. Cytosolic antibodies, Anti-cN1 | a | Neuromuscular | 3 | 3 | 3 | Diagnostic |
| 1062023 | 96 | Which of the following neural tracts play a role in the limbic system? | a. From the anterior nucleus of the thalamus via the fornix b. From the mammillary bodies to the anterior nucleus of the thalamus c. From the hypothalamus to the cingulate gyrus d. From the hypothalamus to the amygdala | b | Miscellaneous | 1 | 2 | 1 | Diagnostic |
| 1062023 | 97 | An 80-year-old man, after a stroke, is able to write a few words but after a few minutes cannot read the words he wrote. Additionally, he cannot name colors but has no impairment in comprehension or speech fluency. Which neurological sign is often associated with this syndrome? | a. Left hand apraxia b. Loss of repetition c. Pure cortical deafness d. Right hemianopsia | d | Vascular Neurology | 2 | 2 | 2 | Diagnostic |
| 1062023 | 98 | An 18-year-old soldier is evaluated for right hand weakness that developed after a stretcher-carrying march. On examination: right hand weakness graded 4/5 without pain, reduced reflexes at all stations, and no pathological reflexes. He reports a similar episode of weakness in the left hand about a month ago. His older brother experienced recurrent limb weakness during military service after lifting heavy objects and was reassigned to an office role. What finding on EMG/NCV would support a diagnosis of genetic neuropathy? | a. Nerve conduction block b. CMAP dispersion c. Electromyographic polyphasic waves d. Uniform slow nerve conduction velocity | d | Genetic Neurology | 2 | 3 | 2 | Diagnostic |
| 1062023 | 99 | A 45-year-old man reports acute onset of vertigo, difficulty speaking, swallowing, and unilateral weakness following a prolonged dental procedure. Which artery is the most common cause of this syndrome? | a. Posterior inferior cerebellar artery b. Vertebral artery c. Basilar artery d. Superior cerebellar artery | b | Vascular Neurology | 2 | 2 | 2 | Diagnostic |
| 1062023 | 106 | What will electrical stimulation of the medial nuclei of the amygdala cause? | a. Pupil constriction and accelerated pulse b. Decrease in motor activity and response to stimuli c. Anger response d. Complex auditory hallucinations | c | Behavioral & Cognitive Neurology | 1 | 2 | 1 | Diagnostic |
| 1062023 | 107 | A 60-year-old woman with a history of diabetes and hypertension, working in accounting, consults a neurologist due to concerns about memory impairment. Her mother developed Alzheimer's disease at the age of 75. She feels she forgets names and events from the past and present. She tends to cry and complains of low energy. Which of the following would support a diagnosis of depression rather than dementia? | a. Attention testing b. Gradual and consistent worsening c. Interviewing family members d. Lack of insight into symptoms | a | Behavioral & Cognitive Neurology | 1 | 2 | 1 | Diagnostic |
| 1062023 | 108 | A 70-year-old man undergoing peritoneal dialysis for end-stage diabetic kidney failure develops progressive limb weakness over two weeks, accompanied by paresthesias in all four limbs. There is no history of fever or vaccination preceding the illness. Nerve conduction studies show significant prolongation of motor and sensory latencies, unevenly distributed, without conduction blocks or dispersion. CSF analysis reveals no cells, protein 55 mg/dL, and normal glucose. There is no significant improvement after two full courses of IVIG. Which of the following could lead to the patient's recovery? | a. Kidney transplantation b. Another course of IVIG c. Hemodialysis d. Steroids | a | Neuromuscular | 2 | 3 | 3 | Therapeutic |
| 1062023 | 109 | A 40-year-old man with a history of migraines since age 16 is hospitalized due to a stroke. His mother has cognitive decline starting at age 60. Brain MRI shows confluent lesions in the periventricular white matter and anterior to the temporal horn. Which gene is involved? | a. NOTCH 3 b. COL4A1 c. HTAR1 gene d. Alpha-galactosidase | a | Genetic Neurology | 2 | 3 | 2 | Diagnostic |
| 1062023 | 110 | A 56-year-old man describes progressive unsteadiness in walking over the past few months, with repeated backward falls without loss of consciousness. He also complains of speech disturbances. On examination, there is impairment of vertical eye movements. What pathological finding is expected on brain MRI? | a. Midbrain atrophy b. Vermis atrophy c. Pons atrophy d. Caudate head atrophy | a | Movement Disorders | 2 | 3 | 2 | Diagnostic |
| 1062023 | 111 | A 28-year-old man, after parathyroid gland resection due to an adenoma, complains of facial spasms, numbness around the mouth, and in the limbs. He later becomes confused with a decline in consciousness. Brain CT is normal. What pathology is expected in lumbar puncture? | a. Pleocytosis b. High protein c. Increased opening pressure d. High lactate | c | Neuromuscular | 2 | 3 | 2 | Diagnostic |
| 1062023 | 112 | A 28-year-old woman arrives at the emergency department after a generalized tonic-clonic seizure. She reports several weeks of fever and headaches. On examination, brain MRI shows multiple T2-hyperintense and T1-hypointense lesions with ring enhancement after gadolinium injection. There is right-sided hemiparesis. HIV test is positive, and CD4+ count is 92 cells/mmÂ³. What is the appropriate treatment at this stage? | a. Chemotherapy b. Highly active antiretroviral therapy (HAART) c. Corticosteroids d. Trimethoprim-sulfamethoxazole | d | Infectious Neurology | 2 | 3 | 2 | Therapeutic |
| 1062023 | 113 | A 67-year-old man is hospitalized due to a subarachnoid hemorrhage from an aneurysm, presenting with headache. He complains of significant worsening of the headache and vomiting. On examination: decreased consciousness, constricted pupils, and upward gaze limitation. What is the most likely cause of his condition? | a. Rebleeding b. Vasospasm c. Hydrocephalus d. Hyponatremia | c | CSF Circulation Disorders | 2 | 3 | 3 | Diagnostic |
| 1062023 | 114 | Which of the following myopathies primarily involves proximal muscles? | a. Myoshi myopathy b. Familial inclusion body myopathy c. Myotonic dystrophy type 2 d. Desmin myopathy | c | Neuromuscular | 1 | 1 | 2 | Diagnostic |
| 1062023 | 117 | A 55-year-old man complains of short episodes of severe pain at the base of the tongue, throat, and ear. There were several episodes of syncope following the pain. What is the most likely diagnosis? | a. Basilar migraine b. Vertebrobasilar TIAs c. Glossopharyngeal neuralgia d. Reflex epilepsy | c | Neuromuscular | 2 | 2 | 2 | Diagnostic |
| 1062023 | 118 | A young female patient describes recurrent episodes of sensory disturbances and burning pain in all four limbs. A similar description exists in her maternal cousin. On examination: areflexia and sensory disturbance in a glove-and-stocking distribution. Workup reveals very low cholesterol levels with significant hypertriglyceridemia. What is the diagnosis? | a. Abetalipoproteinemia b. Tangier Disease c. Fabry disease d. Refsum disease | b | Genetic Neurology | 2 | 3 | 3 | Diagnostic |
| 1062023 | 119 | A 60-year-old man with uncontrolled hypertension presents to the emergency department unconscious after severe headaches and vomiting. On examination: complete quadriplegia, bilateral extensor plantar reflexes, and decerebrate posture. Pupils are constricted but reactive to light. The bleeding is located in the: | a. Pons b. Midbrain c. Medulla oblongata d. Thalamus | a | Vascular Neurology | 2 | 3 | 2 | Diagnostic |
| 1062023 | 120 | Which of the following primary symptoms differentiates parkinsonism from idiopathic Parkinson's disease? | a. Falls b. Rigidity c. Changes in handwriting d. Tremor | a | Movement Disorders | 1 | 2 | 2 | Diagnostic |
| 1062023 | 121 | What pathological findings in the spinal cord are characteristic of HIV infection? | a. Grey matter liquefactive necrosis b. Patchy demyelination c. Wallerian degeneration of descending tracts d. White matter vacuolization | d | Infectious Neurology | 1 | 2 | 1 | Diagnostic |
| 1062023 | 122 | A 75-year-old man presents with complaints of gait disturbance developing over the past year. Additionally, in recent months, he has experienced memory decline and intermittent urinary incontinence. Brain MRI shows significant generalized ventricular enlargement with mild peripheral atrophy. Lumbar puncture reveals an opening pressure of 120 mm H2O. Which of the following findings would support the decision to insert a ventriculoperitoneal shunt? | a. Opening pressure on lumbar puncture of 120 mm H2O b. Presence of focal findings on neurological examination c. Improvement in gait after lumbar puncture d. Presence of significant dementia | c | CSF Circulation Disorders | 2 | 3 | 3 | Therapeutic |
| 1062023 | 123 | A 40-year-old engineer presents with proximal muscle weakness that began in recent months. The patient describes a history of hand myotonia since childhood. Additionally, he underwent cataract surgery at a young age. What is the genetic disorder in this patient? | a. 200 repeats of CTG in the DMPK gene b. Other genetic mutation c. Another genetic disorder d. None of the above | b | Genetic Neurology | 2 | 3 | 2 | Diagnostic |
| 1062023 | 124 | A 45-year-old man describes a two-month history of gait instability and ophthalmoplegia. Blood tests revealed anti-Ma antibodies. Which tumor is most commonly associated with these antibodies? | a. Lymphoma b. Testicular tumor c. Lung tumor d. Renal tumor | b | Neuro-oncology | 2 | 2 | 2 | Diagnostic |
| 1062023 | 125 | A 45-year-old healthy man complains of heaviness, tingling, and discomfort in his legs during sleep. He must move his legs to relieve the symptoms, which are worse in the summer. A deficiency of which metal could cause this condition? | a. Zinc b. Magnesium c. Calcium d. Iron | d | Neuromuscular | 1 | 1 | 2 | Diagnostic |
| 1062023 | 126 | A 55-year-old man presents with facial and head pain, subfebrile fever for two days. Examination reveals no meningeal signs, limited abduction in the right eye, and sensory disturbance in all three branches of the right trigeminal nerve. No limb weakness is noted. CRP is elevated. What is the most likely diagnosis? | a. Jugular sinus thrombosis b. Cavernous sinus granuloma c. Petrositis d. Parotitis | c | Infectious Neurology | 2 | 3 | 2 | Diagnostic |
| 1062023 | 127 | A 30-year-old man suffers from oral and genital aphthae and arthritis. Which of the following is NOT a typical neurological manifestation of his disease? | a. Polyradiculopathy b. Venous thrombosis of cerebral veins c. Sixth cranial nerve palsy d. Meningoencephalitis | a | Neuroimmunology | 2 | 2 | 2 | Diagnostic |
| 1062023 | 128 | A 20-year-old man has had difficulty performing sports activities, instability in standing and walking since childhood. Recently, he developed difficulty with fine motor tasks and unclear speech. Examination reveals dysarthric speech, nystagmus, sensory ataxia, and cerebellar signs. Which of the following proteins is defective? | a. Merlin b. Frataxin c. Huntingtin d. Hamartin | b | Genetic Neurology | 2 | 3 | 2 | Diagnostic |
| 1062023 | 129 | A 35-year-old vegetarian woman, self-medicating with vitamin and mineral supplements, recently developed paresthesias in her limbs and face and instability in walking. Examination reveals sensory ataxia and areflexia, with normal strength and no pyramidal signs. What is the most likely cause of her condition? | a. Excess thiamine b. Excess copper c. Excess pyridoxine d. Excess zinc | c | Neuromuscular | 2 | 2 | 2 | Diagnostic |
| 1062023 | 130 | What is the most common type of polyneuropathy in patients with HIV infection? | a. Painful mononeuropathy multiplex b. Distal, symmetrical, axonal polyneuropathy c. Subacute inflammatory cauda equina syndrome d. Inflammatory demyelinating peripheral neuropathy | b | Neuromuscular | 1 | 1 | 1 | Diagnostic |
| 1062023 | 131 | A 31-year-old man with gradually progressive weakness in the pelvic and shoulder girdles. CK levels are elevated fivefold above normal. On examination, bilateral Achilles tendon contractures were reported early in the disease. Which protein is defective? | a. Dysferlin b. Dystrophin c. Calpain d. Sarcoglycan | c | Neuromuscular | 2 | 3 | 2 | Diagnostic |
| 1062023 | 132 | Which of the following statements regarding the type of cells affected in neuromyelitis optica (NMO) compared to multiple sclerosis (MS) is correct? | a. In NMO, the primary damage is to microglia, whereas in MS, the damage is to astrocytes. b. The commonality between NMO and MS is that in both diseases, the affected neural cells are oligodendrocytes. c. In NMO, the primary damage is to astrocytes, whereas in MS, the damage is to oligodendrocytes. d. In NMO, the primary damage is to microglia, whereas in MS, the damage is to oligodendrocytes. | c | Neuroimmunology | 1 | 2 | 2 | Diagnostic |
| 1062023 | 133 | A 20-year-old man complains of 'confusion.' He describes visual hallucinations, delusions, and incoherent speech. On examination, his attention is relatively preserved, and his orientation is intact. What is the most appropriate cause of his condition? | a. Alcohol withdrawal b. Acute bacterial septicemia c. Psychotic disorder d. Hypoglycemia | c | Behavioral & Cognitive Neurology | 1 | 1 | 2 | Diagnostic |
| 1062023 | 134 | A 19-year-old man complains of recurrent episodes of inability to move his limbs upon waking in the morning. These episodes usually resolve within a few minutes. Recently, the frequency of these episodes has significantly increased. Which treatment can alleviate this condition? | a. Brotizolam b. Clomipramine c. Ropinirole d. Clonazepam | b | Behavioral & Cognitive Neurology | 1 | 3 | 2 | Therapeutic |
| 1062023 | 135 | A 50-year-old man complains of facial swelling and difficulty whistling. On examination, there is bilateral facial muscle weakness, bilateral swelling of the salivary glands, and an elevated erythrocyte sedimentation rate. What is the most likely diagnosis? | a. Sarcoidosis b. Guillain-Barre Syndrome c. Bilateral Bell's palsy d. Melkersson-Rosenthal syndrome | a | Neuroimmunology | 2 | 2 | 2 | Diagnostic |
| 1062023 | 136 | A 45-year-old woman with a history of breast cancer has been experiencing headaches with vomiting for two weeks. This morning, her family noticed right-sided body weakness and speech disturbances. Occlusion of which vein would lead to this clinical presentation? | a. Sagittal sinus b. Cavernous sinus c. Vein of Galen d. Straight sinus | a | Vascular Neurology | 2 | 2 | 2 | Diagnostic |
| 1062023 | 137 | A patient with idiopathic Parkinson's disease receiving various symptomatic medications complains of significant fatigue and sudden episodes of falling asleep during the day. The patient has also started gambling excessively. Which of the following medications could cause these symptoms? | a. Amantadine b. Carbidopa-L-dopa c. Ropinirole d. Rasagiline | c | Movement Disorders | 1 | 2 | 2 | Diagnostic |
| 1062023 | 138 | A 37-year-old male with a history of schizophrenia treated with haloperidol is found confused in his apartment. On examination: drowsy, responsive to painful stimuli, fever of 39.0Â°C, sweating, no focal neurological signs, and rigid tone. What is the appropriate treatment for his condition? | a. Flumazenil b. Cyproheptadine c. Dantrolene d. Olanzapine | c | Movement Disorders | 1 | 3 | 2 | Therapeutic |
| 1062023 | 139 | Which neurological clinical manifestation of syphilis can be seen in the early stage of the disease? | a. General paresis b. Meningovascular disease c. Syphilitic amyotrophy d. Syphilitic cerebellar ataxia | b | Infectious Neurology | 1 | 1 | 1 | Diagnostic |
| 1062023 | 140 | A 23-year-old woman with a history of obesity presents with three months of headaches, blurred vision, and horizontal diplopia with transient episodes of vision loss. On examination: papilledema without focal findings. Brain imaging shows no evidence of a space-occupying lesion or venous thrombosis. Lumbar puncture reveals an opening pressure of 370 mm H2O with normal cerebrospinal fluid content. All the following findings are typical in visual field testing for this condition except: | a. Enlargement of the blind spot b. Peripheral constriction of the visual field c. Temporal field defect d. Inferior nasal field defect | c | CSF Circulation Disorders | 2 | 3 | 2 | Diagnostic |
| 1062023 | 141 | A 20-year-old soldier complains of severe muscle pain and diffuse muscle weakness after a long march conducted a day after fasting for Purim. His urine appears cola-colored. In the past, he has had episodes of muscle weakness that did not improve with rest. What is the most likely diagnosis? | a. McArdle disease (type V Glycogenosis) b. Tarui disease (type VII Glycogenosis) c. Carnitine Palmitoyltransferase Deficiency (type II) d. Phosphoglycerate Kinase deficiency | a | Genetic Neurology | 2 | 3 | 2 | Diagnostic |
| 1062023 | 142 | Which of the following tumors is characterized by a more benign course over time (lower grade)? | a. Oligodendroglioma (IDH-mutant and 1p/19q co-deletion) b. Dysembryoplastic neuroepithelial tumor c. Ependymoma d. Diffuse astrocytoma (IDH mutant) | b | Neuro-oncology | 1 | 2 | 1 | Diagnostic |
| 1062023 | 143 | A 70-year-old male with advanced Parkinson's disease and motor fluctuations is hospitalized due to confusion and restlessness with disorientation. Which medication adjustment might alleviate the symptoms of restlessness and confusion? | a. Rapid reduction of levodopa b. Rapid reduction of quetiapine c. Rapid reduction of biperiden d. Rapid reduction of ropinirole | d | Movement Disorders | 2 | 2 | 3 | Therapeutic |
| 1062023 | 144 | A 70-year-old male with Parkinson's disease. His wife complains that he shouts and moves his arms and legs aggressively during sleep. Which of the following medications is the treatment of choice for this problem? | a. Amitriptyline b. Quetiapine c. Melatonin d. Bupropion | c | Movement Disorders | 1 | 2 | 2 | Therapeutic |
| 1062023 | 145 | A 40-year-old male with a history of right-sided peripheral facial palsy presents to the clinic due to short, uncontrolled facial muscle spasms on the right side. On examination, brief, non-rhythmic spasms of the right facial muscles are prominent. Which treatment can alleviate this condition? | a. Valproic acid b. Botulinum toxin injections c. Clonazepam d. Gabapentin | b | Neuromuscular | 1 | 2 | 2 | Therapeutic |
| 1062023 | 146 | Which clinical manifestation is not seen in a stroke of the anterior choroidal artery? | a. Hemiparesis b. Sensory disturbances c. Hemianopia d. Motor aphasia | d | Vascular Neurology | 1 | 2 | 1 | Diagnostic |
| 1062023 | 147 | A 70-year-old male has had recurrent episodes of violence towards his wife during sleep over the past year. Additionally, he has significant slowness in walking, with a stooped gait and small steps, and resting tremor in the right hand. Every few days, he experiences recurrent episodes of confusion and significant agitation accompanied by visual hallucinations. Each episode lasts several hours and occurs mainly in the evening. Which medication is recommended to treat the agitation and visual hallucinations? | a. Diazepam b. Rivastigmine c. Haloperidol d. Biperiden | b | Movement Disorders | 2 | 3 | 3 | Therapeutic |
| 1062023 | 148 | A 15-year-old male was brought to the hospital due to vomiting, loss of appetite, followed by generalized weakness, blurred vision, difficulty speaking, and swallowing. On examination: fully conscious, nasal speech, bilateral ptosis, limitation of eye movements in all directions, dilated pupils unresponsive to light and accommodation, flaccid limb weakness, and no sensory disturbances. It was later revealed that other family members experienced similar symptoms in recent days. As part of the evaluation, a nerve conduction study was performed. What findings are expected? | a. Slowing of motor conduction b. Conduction block c. Low motor amplitudes d. Prolonged F waves | c | Neuromuscular | 2 | 3 | 3 | Diagnostic |
| 1062023 | 149 | A patient presents with paralysis of cranial nerves 9, 10, 11, and 12, along with Horner's syndrome. Which of the following is the likely site of the lesion? | a. Pontocerebellar angle b. Apex of petrous bone c. Jugular foramen d. Posterior retroparotid space | c | Neurophthalmology | 2 | 2 | 2 | Diagnostic |
| 1062023 | 150 | A young patient, after left-sided facial muscle paralysis a few months ago, began complaining of mild contractions in the left cheek every time he blinks or closes his left eye. What is this phenomenon called? | a. Synkinesis b. Hemifacial spasm c. Facial myokymia d. Myotonia | a | Neuromuscular | 2 | 1 | 2 | Diagnostic |
| 1062023 | 39 | A 30-year-old woman presents with decreased vision in her right eye and eye pain. An eye examination reveals a macular star. What is the most likely cause of this condition? | a. Bartonella henselae b. Cytomegalovirus c. Plasmodium malariae d. Treponema pallidum | a | Neurophthalmology | 2 | 2 | 2 | Diagnostic |
| 1062023 | 40 | Bilateral damage to which area of the brain may cause systemic fever? | a. Hypothalamus in the optic preoptic area b. Locus ceruleus c. Caudate nucleus d. VIM nucleus in the thalamus | a | Behavioral & Cognitive Neurology | 1 | 1 | 1 | Diagnostic |
| 1062023 | 41 | Arnold-Chiari malformation is characterized by: | a. Cerebellar ataxia b. Orthostatic headache c. Impairment of posterior columns d. Horizontal nystagmus with direction changes | a | CSF Circulation Disorders | 1 | 1 | 1 | Diagnostic |
| 1062023 | 42 | A 66-year-old man presents with headaches, seizures, and cognitive impairment for six months. Brain MRI shows a large space-occupying lesion, and biopsy reveals hypercellularity, atypia, nuclear fibrillary astrocytes combined with primitive cells, giant cells, numerous mitoses, necrosis, and hemorrhage. What characterizes this tumor? | a. High prevalence in the posterior fossa b. The tumor is mostly multicentric c. The tumor does not involve the corpus callosum d. Extracranial metastases are rare | d | Neuro-oncology | 2 | 2 | 2 | Diagnostic |
| 1062023 | 44 | A 40-year-old man presents with right-sided drop foot. Examination reveals no neurological signs in the upper limbs. In the lower limbs, there is weakness in dorsiflexion, eversion, and inversion of the right foot. Reflexes are symmetrical. Sensory disturbance is noted along the right shin. What is the most likely diagnosis? | a. L5 radiculopathy b. Peroneal nerve palsy c. Sciatic nerve palsy d. Tibial nerve palsy | a | Neuromuscular | 2 | 3 | 2 | Diagnostic |
| 1062023 | 45 | A patient presents with upward gaze palsy, retraction nystagmus, and eyelid retraction. Where is the lesion located? | a. Dorsal midbrain b. Pons c. Medial longitudinal fasciculus d. Neuromuscular junction | a | Neurophthalmology | 2 | 2 | 2 | Diagnostic |
| 1062023 | 100 | A 21-year-old man has been complaining for two days of unsteady gait and binocular diplopia. Neurological examination reveals restricted eye movements in all directions and areflexia. Which of the following is likely to be found in blood tests? | a. Antibodies to voltage-gated calcium channels b. Antibodies to GQ1B c. Antibodies to acetylcholine receptor d. Antibodies to thyroid peroxidase | b | Neuromuscular | 2 | 2 | 2 | Diagnostic |
| 1062023 | 101 | A 27-year-old woman, 10 weeks pregnant, suffers from excessive vomiting. For two days, she has been complaining of unsteady gait, blurred vision, and difficulty concentrating. What ophthalmological finding is expected in this patient? | a. Internuclear ophthalmoplegia b. Bilateral ptosis c. Miotic pupils d. Restricted eye abduction | d | Neurophthalmology | 2 | 2 | 2 | Diagnostic |
| 1062023 | 102 | What causes damage in malaria infection of the central nervous system? | a. Arachnoiditis b. Direct neuronal damage c. Demyelination d. Capillary obstruction | d | Infectious Neurology | 1 | 2 | 1 | Diagnostic |
| 1062023 | 103 | A 23-year-old woman with a history of tension-type headaches is treated with amitriptyline for prevention. She presents to the emergency department with palpitations. ECG shows evidence of tachyarrhythmia. Which of the following findings supports amitriptyline overdose? | a. Excessive sweating b. Drooling c. Pupil dilation d. Ptosis | c | Behavioral & Cognitive Neurology | 1 | 2 | 2 | Diagnostic |
| 1062023 | 38 | A 30-year-old man suffers from dystonia, choreiform movements, recurrent tongue biting, and vocal tics. Cognitive evaluation shows widespread impairment. Which of the following tests is pathological in this case? | a. Acanthocytes on blood smear b. Serum ceruloplasmin level c. CSF LGI1 autoantibodies d. High serum cholestanol levels | a | Genetic Neurology | 2 | 2 | 2 | Diagnostic |
| 1062023 | 56 | A 67-year-old woman with diabetes. Suffering for 5 years from dry eyes and mouth, joint pain, and progressive burning sensation in feet and calves. Examination: normal strength, mild decrease in pain and temperature sensation in calves and feet. Normal touch, vibration, and position sense. Normal reflexes. Normal nerve conduction study. The patient's prominent nerve damage is expected to be in nerve fibers ending in- | a. Meissner corpuscles b. Pacini corpuscles c. Free nerve endings d. Ruffini plumes | c | Neuromuscular | 2 | 3 | 2 | Diagnostic |
| 1062023 | 57 | A 32-year-old man underwent trauma with complete transection of the thoracic spinal cord at T10 level. Which of the following findings is expected in the acute phase? | a. Tetraplegia b. Loss of sensation from feet to nipple level c. Increased blood pressure d. Urinary retention | d | Miscellaneous | 1 | 1 | 1 | Diagnostic |
| 1062023 | 59 | What is the most common somatic (acquired) genetic finding in meningiomas? | a. Mutation in VEGF gene b. Truncating mutation in Merlin (NF2) gene c. Mutations in Estrogen receptor gene d. Deletions in chromosome 1p | b | Neuro-oncology | 1 | 1 | 1 | Diagnostic |
| 1062023 | 60 | An 80-year-old woman one week after left knee replacement surgery, with pain in the surgical area. She was admitted after a stroke. On examination: when touching her left knee she identifies the painful leg. When simultaneously stimulating both knees she feels pain but reports to the examiner that he only touched the right knee. What is the location of the infarct? | a. Pons b. Post central gyrus c. Posterior limb internal capsule d. Thalamus | b | Vascular Neurology | 2 | 3 | 2 | Diagnostic |
