# Supplementary material for: A multi-agent approach to neurological clinical reasoning
Source: PLOS Digit Health. 2025 Dec 4;4(12):e0001106. doi: 10.1371/journal.pdig.0001106 (PMC12677565; doi:10.1371/journal.pdig.0001106)
Supplement: S3 File — (DOCX) [file pdig.0001106.s003.docx]

S3 File.

**Systematic Error Analysis of Multi-Agent Framework Performance on Six Representative Questions Demonstrating Retrieval Failures and Reasoning Failures Across Different Neurological Subspecialties and Complexity Levels.**

**Example 1:** Question 1052024-33

**Complexity scores:** FKD 3, CCI 3, RC 2

**The question:** A 20-year-old man experiences recurring episodes of ataxia and dysarthria lasting several minutes and resolving. Neurological examinations show periorbital muscle rippling (muscle rippling) between attacks. Common trigger for these episodes is physical activity or startle. Similar symptoms are found in his mother. Which of the following treatments is NOT suitable as preventive treatment for this condition?

**Choices:**a. 4-aminopyridine

b. Acetazolamide

c. Benzodiazepines

d. Carbamazepine

**Correct Answer:** c

**Framework Answer:** a

**Error type:** Reasoning failure - ignored explicit evidence and retrieval gap

**Analysis:** The multi-agent framework retrieved a passage explicitly stating "Carbamazepine or 4-aminopyridine are beneficial in some patients" in the context of episodic ataxia treatment. Despite this clear evidence, the synthesis agent concluded that 4-aminopyridine is unsuitable for EA1 prophylaxis, reasoning that it is "more effective in episodic ataxia type 2." This directly contradicts the retrieved text which describes 4-aminopyridine as beneficial without restricting it to EA2 only. Meanwhile, the retrieved passages mentioned benzodiazepines only in the context of hyperekplexia treatment ("Effective prevention with clonazepam or valproate") and as a general drug class causing sedation, but never discussed benzodiazepines as prophylactic treatment for episodic ataxia attacks. The validation loop reinforced the error by accepting reasoning based on assumed EA1 vs EA2 treatment distinctions that were not supported by the retrieved text. This represents the same reasoning failure pattern seen earlier: ignoring explicit evidence stating a medication is beneficial while failing to recognize that the correct answer (benzodiazepines) lacked any supporting evidence for use in EA prophylaxis.

**Example 2:** Question 1052024-58

**Complexity scores:** FKD 1, CCI 3, RC 2

**The Question:** A 72-year-old man with Parkinson's disease complains of excessive daytime sleepiness. He is treated with Levodopa. He has symptoms of REM sleep behavior disorder (RBD), restless legs syndrome (RLS), and his sleep is disrupted due to frequent awakenings (insomnia). Which of these findings in the description is NOT related to causing excessive daytime sleepiness in the patient?

**Choices:**
a. Levodopa

b. Insomnia

c. RBD-REM sleep behavior disorder

d. RLS - restless legs syndrome

**Correct Answer:** c

**Framework Answer:** a

**Error type:** Reasoning failure - Ignored explicit evidence

**Analysis:** The multi-agent framework retrieved multiple explicit passages stating that dopaminergic medications are the primary cause of excessive daytime sleepiness in Parkinson's disease, with the text repeatedly emphasizing "In most cases, this excessive daytime drowsiness is related to dopaminergic drugs" and "daytime hypersomnolence and irresistible sleep attacks...may be due to a combination of the intrinsic disease process and dopaminergic medications." Despite this unambiguous evidence, the framework selected Levodopa as NOT causing EDS, reasoning that "Levodopa itself is generally less likely to be a direct driver...compared to dopamine agonists." This represents a fundamental misreading of the question's clinical reality versus the retrieved evidence. The framework failed to recognize that while the retrieved passages mention RBD, insomnia, and RLS as common in PD, they never explicitly state that RBD CAUSES excessive daytime sleepiness - RBD is described as a sleep disorder associated with PD, but the causal link to daytime somnolence is not established in the retrieved text. Meanwhile, the passages definitively state that dopaminergic drugs (which includes Levodopa) are "in most cases" the cause of EDS in PD patients. The validation loop compounded this error by accepting reasoning based on drug class distinctions (Levodopa vs dopamine agonists) while ignoring that the retrieved evidence made no such distinction and explicitly identified dopaminergic medications broadly as causing EDS.

**Example 3:** Question 1062024-52

**Complexity scores:** FKD 2, CCI 2, RC 2

**The Question:** A 77-year-old man with a history of smoking and hypertension has been experiencing vomiting and diarrhea in recent days. He arrives at the emergency department due to sudden onset weakness in the right side of the body, involving the shoulder and thigh more than the face. What is most likely to be found in the CTA of the neck and brain?

**Choices:**
a. Occlusion of the left MCA

b. Severe stenosis of the left ICA

c. Occlusion of the left ACA

d. Dissection of the left vertebral artery

**Correct Answer:** c

**Framework Answer:** b

**Error type:** Reasoning failure - narrative distraction and pattern mismatching

**Analysis:** The multi-agent framework retrieved a passage that precisely described the clinical presentation: "Anterior cerebral artery...Infarction in the ACA distribution produces contralateral leg weakness. The arm may be slightly affected, especially the proximal arm, with sparing of hand and face." This is an exact match for the question's description of right-sided weakness involving shoulder and thigh more than face. However, the framework became distracted by constructing a sophisticated narrative integrating the dehydration context (vomiting/diarrhea) with watershed infarct pathophysiology, reasoning that hypovolemia plus ICA stenosis caused border-zone ischemia presenting as "man-in-a-barrel" syndrome. This reasoning had three critical flaws: (1) man-in-a-barrel syndrome is classically BILATERAL proximal arm weakness, not unilateral hemiparesis as described in the question, (2) the framework prioritized integrating contextual details over pattern-matching the symptom distribution, and (3) it ignored retrieved text explicitly describing ACA territory infarcts as causing exactly this presentation. The validation loop reinforced this error by praising the "precise clinical rationale" without verifying whether the clinical presentation actually matched watershed patterns versus ACA territory. This represents narrative distraction where the framework's attempt to create an intellectually satisfying synthesis of all clinical details (age, risk factors, dehydration, stroke) led it to construct an overly complex explanation while missing the straightforward match between the symptom pattern and the retrieved ACA territory description.

**Example 4**: Question 1062023-141

**Complexity scores:** FKD 2, CCI 3, RC 2

**The Question:** A 20-year-old soldier complains of severe muscle pain and diffuse muscle weakness after a long march conducted a day after fasting for Purim. His urine appears cola-colored. In the past, he has had episodes of muscle sweakness that did not improve with rest. What is the most likely diagnosis?

**Choices:**
a. McArdle disease (type V Glycogenosis)

b. Tarui disease (type VII Glycogenosis)

c. Carnitine Palmitoyltransferase Deficiency (type II)

d. Phosphoglycerate Kinase deficiency

**Correct Answer:** a

**Framework Answer:** c

**Error Type:** Reasoning failure - narrative distraction

**Analysis:** The multi-agent framework retrieved comprehensive information about metabolic myopathies including explicit passages describing both CPT II deficiency ("Typically, CPT deficiency manifests with myoglobinuria after strenuous exercise... Affected individuals are particularly predisposed to these attacks if exercise occurs in the fasting state") and McArdle disease ("exercise intolerance experienced pain and tightness of his muscle on forced exercise"). However, the framework anchored on the mention of "fasting for Purim" and constructed a narrative around CPT II deficiency as a disorder of fatty acid oxidation that worsens with fasting. This led the framework to over-weight a contextual detail (brief religious fast) while missing that the core presentation—young person with recurrent exercise-induced myoglobinuria and cola-colored urine—is the textbook description of McArdle disease, the most common glycogen storage disorder causing exercise intolerance. The framework also misinterpreted "episodes that did not improve with rest," using this to support CPT II rather than recognizing it as describing the persistent/recurrent nature of attacks rather than absence of second-wind phenomenon. The validation loop reinforced this error by confirming that all clinical features matched CPT II without questioning whether fasting on Purim (a brief daytime fast) would truly be sufficient to trigger a fatty acid oxidation disorder, or whether the classic triad of exercise intolerance + myoglobinuria + young adult more strongly suggested the far more common McArdle disease. This represents narrative distraction where the framework fixated on one clinical detail to construct a sophisticated but incorrect diagnostic story, while failing to recognize the pattern-match to the most prevalent condition presenting with these exact symptoms.

**Example 5:** Question 1062023-143

**Complexity scores:** FKD 2, CCI 2, RC 3

**The Question:** A 70-year-old male with advanced Parkinson's disease and motor fluctuations is hospitalized due to confusion and restlessness with disorientation. Which medication adjustment might alleviate the symptoms of restlessness and confusion?

**Choices:**
a. Rapid reduction of levodopa

b. Rapid reduction of quetiapine

c. Rapid reduction of biperiden

d. Rapid reduction of ropinirole

**Correct Answer:** d

**Framework Answer:** c

**Error type:** Reasoning failure - narrative distraction and ignored explicit evidence

**Analysis:** The multi-agent framework retrieved highly relevant information about managing confusion in Parkinson's disease, including passages explicitly stating that "anticholinergics such as benztropine and trihexyphenidyl can cause confusion" and recommending discontinuation of medications with anticholinergic properties. Based on this, the framework concluded that reducing biperiden (an anticholinergic) would alleviate confusion, applying the well-established principle that anticholinergics exacerbate delirium in elderly patients. However, the framework failed to adequately process a critical retrieved passage stating: "Discontinuation of anticholinergics, selegiline, and amantadine before reducing l-dopa is recommended. Following these discontinuations, reduction and simplification of dopamine agonists may be beneficial." This passage discusses dopamine agonists as contributors to psychotic symptoms in PD and suggests their reduction as part of symptom management. The framework also retrieved information that "dopamine agonists may contribute to the development of psychosis" but underweighted this evidence. The reasoning failure occurred because the framework anchored on a strong general heuristic (anticholinergics = confusion in elderly) without properly weighing that: (1) dopamine agonists are well-known causes of psychosis and confusion in PD patients, (2) in advanced PD with motor fluctuations, ropinirole (a dopamine agonist) might be more dispensable than maintaining other dopaminergic support, and (3) the clinical context of "advanced PD with motor fluctuations" should have triggered consideration of dopaminergic medication side effects as a primary differential. The validation loop reinforced this error by confirming the anticholinergic hypothesis without questioning whether other retrieved evidence pointed to dopamine agonists as equally or more likely culprits. This represents overgeneralization of a valid clinical principle while ignoring context-specific reasoning and alternative evidence present in the retrieved text suggesting dopamine agonist reduction as appropriate management for PD-associated confusion.

**Example 6:** Question 1092024-32

**Complexity scores:** FKD 2, CCI 3, RC 3

**The Question:** A 20-year-old man with recurrent episodes of significant limb weakness lasting about an hour and resolving. Neurological examination reveals difficulty opening the fist after strong and prolonged muscle contraction. In a muscle exercise test, the CMAP amplitude on EMG increases with each effort. However, in a prolonged exercise test, there is a temporary increase in amplitude followed by a prolonged decrease lasting 40 minutes. What is the diagnosis?

**Choices:**
a. Hyperkalemic periodic paralysis

b. Hypokalemic periodic paralysis

c. Myotonia congenita

d. Paramyotonic congenita

**Correct Answer:** a

**Framework Answer:** d

**Error type:** Reasoning failure - over-integration

**Analysis:** The framework retrieved text explicitly stating: "The predominant symptom in patients with hyperkalemic periodic paralysis is weakness...Myotonia may be present...but the predominant difficulty is recurrent bouts of paralysis. The allelic disease paramyotonia congenita causes muscle stiffness, and bouts of weakness are mild." The question presents recurrent episodes of significant limb weakness as the primary complaint, with myotonia as an associated finding. The framework correctly identified the Fournier Pattern I EMG finding (prolonged CMAP amplitude decrease lasting 40 minutes) and matched it to paramyotonia congenita. However, this represents over-integration: the framework forced all three findings—weakness episodes (primary complaint), myotonia (secondary finding), and EMG pattern—into a single diagnosis (paramyotonia) when the retrieved text explicitly distinguished these allelic disorders by which symptom predominates. The question states weakness is "significant" and recurrent, which the retrieved text identifies as characteristic of hyperKPP ("predominant difficulty is recurrent bouts of paralysis"), whereas paramyotonia has "mild" weakness with stiffness as the dominant feature. Both conditions can show myotonia and share EMG abnormalities, but clinical diagnosis depends on symptom hierarchy. The framework became anchored on the EMG pattern match without recognizing that the primary presenting complaint (recurrent significant weakness episodes) determines the diagnosis when distinguishing between allelic channelopathies with overlapping features.
