# Supplementary material for: A multi-agent approach to neurological clinical reasoning
Source: PLOS Digit Health. 2025 Dec 4;4(12):e0001106. doi: 10.1371/journal.pdig.0001106 (PMC12677565; doi:10.1371/journal.pdig.0001106)
Supplement: Attachment — Submitted filename: MultiAgent_PLOS - review response.pdf [file pdig.0001106.s005.pdf]

Editor comments (synthesis all three reviews):

COMMENT: 1. Highlight early the MedQA discrepancies: Make clear in the Abstract or early Results that RAG improved board-style performance but sometimes reduced MedQA accuracy (e.g., LLaMA 3.3-70B, o1 slight drop). Then cross-reference your explanation that the RAG knowledge base was tailored to board questions rather than MedQA. Add a one-sentence rationale in the RAG Framework subsection of Methods noting this domain shift (currently discussed later).

RESPONSE: We thank the reviewer for this important observation. We agree that the divergent RAG effects across datasets warrant early highlighting and explicit explanation. We have made the following changes:

Abstract - we added a new sentence immediately after reporting the LLaMA 3.3-70B agentic results that explicitly states:

> *"External validation on MedQA revealed dataset-specific RAG effects: while RAG improved board certification performance, it showed minimal benefit on MedQA questions (LLaMA 3.3-70B: +1.4% vs +3.9% on board exams), reflecting alignment between our specialized neurology textbook and board examination content rather than the broader medical knowledge required for MedQA."*

Discussion - we expanded the RAG discussion to contextualize these findings within the broader implications for clinical AI systems:

> *"Furthermore, the divergent RAG effects between board certification questions (consistent improvements) and MedQA questions (minimal or negative impact) highlight a critical consideration: RAG performance depends heavily on alignment between the knowledge base and target assessment domain. Our specialized neurology textbook enhanced reasoning on neurological board examinations but provided limited value for MedQA's broader medical questions, indicating that clinical AI systems may require multiple, complementary knowledge bases to handle the full spectrum of medical reasoning tasks encountered in practice."*

COMMENT: 2. Hyperparameters and tuning: Methods state standardized prompts (temperature 0.7, top-p 0.9) for all models. Please justify this choice and state explicitly whether any model-specific tuning/ablation was attempted. If none, say so; if yes, report the grid/ranges tried and the selection rule.

RESPONSE: We thank the reviewer for raising this important point. In medical question answering, it is important to prioritize accuracy and reduce hallucinations, so temperatures below or around 0.7 are often chosen to maintain reliability while allowing some flexibility in responses. While a lower temperature like 0 or 0.3 may maximize reproducibility and reliability, 0.7 is still low enough to avoid incoherence and major drops in performance but can introduce a

slightly more natural and less deterministic feel in the answer style. A temperature of 0.7 falls within this stable range, striking a balance between creativity and factual accuracy. Top-p (nucleus sampling) is a parameter that limits the pool of possible next words by summing their probabilities until 90% of the total probability mass is reached. This approach allows for both a temperature-controlled level of creativity and a controlled vocabulary by cutting off less likely words, resulting in a more coherent and less random output than using temperature alone. We follow recent publications that use similar parameters, including Renze & Guven, arXiv 2024, Windisch et al. Cureus 2024.

In the Methods - Experiments Evaluation section we now report:

> *"We selected temperature 0.7 to balance factual accuracy with response naturalness, following established practices in medical question answering [24,25], where temperatures ≤0.7 minimize hallucinations while maintaining response quality. Top-p 0.9 further constrains sampling to high-probability tokens, enhancing output coherence. No model-specific hyperparameter tuning was performed to ensure fair cross-model comparison."*

COMMENT: 3. Error analysis: Please expand briefly: categorize a few representative error types (e.g., genuinely ambiguous stems, underspecified vignettes, low-consensus items, distractor confusability) and provide 2–3 concise exemplars (ideally one L3/CCI, one L3/FKD). Indicate whether agent validation loops failed due to retrieval gaps vs. reasoning slips.

RESPONSE: We thank the reviewer for this valuable suggestion to systematically categorize error types and examine validation loop failures. We have added a new section "Error Analysis of Multi-Agent Framework" in the Results section:

> *'To understand the limitations of our multi-agent framework, we conducted a systematic analysis of the remaining errors made by the top-performing model. The framework's errors fall into two primary categories: retrieval failures (RAG gaps where relevant information was not retrieved from the knowledge base) and reasoning failures (where retrieved information was present but misused or ignored). Within reasoning failures, we identified four distinct patterns: (1) ignored explicit evidence - correct answers existed in retrieved text but were disregarded in favor of alternative reasoning; (2) narrative distraction - construction of sophisticated but incorrect explanations instead of matching straightforward clinical patterns; (3) over-integration - forcing disparate clinical findings into a single diagnosis when they belonged to different conditions; and (4) pattern mismatching - failure to connect clinical descriptions in questions to corresponding descriptions in retrieved passages.*
>
> *Critically, validation loops failed differently across error types. For retrieval gaps, validation agents had no mechanism to detect missing information, as they could only assess the coherence of reasoning based on available evidence. For reasoning failures, validation loops often reinforced initial errors rather than catching them, suggesting the quality assurance agent shared the same reasoning blind spots as the synthesis agent,*

both prioritizing internal narrative coherence over strict textual fidelity to retrieved evidence. Representative examples demonstrating each error pattern with detailed analysis are provided in Supplement 3."

We created a new **Supplement 3**: Systematic Error Analysis of Multi-Agent Framework Performance on Six Representative Questions Demonstrating Retrieval Failures and Reasoning Failures Across Different Neurological Subspecialties and Complexity Levels. This supplement includes detailed analysis of six representative examples spanning different complexity levels. For each example, we provide the complete question, complexity scores, error type classification, thorough analysis of the failure mechanism, and key excerpts from retrieved passages and agent reasoning showing where the breakdown occurred.

COMMENT: 4. Clinical vs. therapeutic reasoning: Please clarify how the multi-agent approach maps onto neurological clinical reasoning (localization, syndrome synthesis) and therapeutic reasoning (management choices). Quantify what fraction of items involved management/therapy, and add one worked example tracing agents' roles for a management-focused question.

RESPONSE:

We thank the reviewer for highlighting the importance of distinguishing between clinical reasoning (diagnosis, localization, syndrome synthesis) and therapeutic reasoning (management and treatment decisions). We added a "Reasoning Type" classification column to **Supplement 2** - Table S1: Three-Dimensional Complexity Classification of 305 Neurological Board Certification Questions by Factual Knowledge Depth, Clinical Concept Integration, Reasoning Complexity and Reasoning Type), categorizing each question as either "Diagnostic" or "Therapeutic."

In the results section, we now report:

*"We classified each question by reasoning type: 263 questions (86%) required diagnostic reasoning such as syndrome identification, localization, and differential diagnosis, while 42 questions (14%) required therapeutic reasoning including treatment selection and management approaches (**Supplement 2: S1 Table**)."*

To illustrate how the multi-agent framework maps onto neurological clinical reasoning for management-focused questions, we added Box 1 (Step-by-Step Analysis of Multi-Agent Framework Performance on a Complex Clinical Question) in the Results section (page 12), with the following text:

"To illustrate how the multi-agent architecture handles therapeutic reasoning, Box 1 presents a representative example involving treatment selection for uremic neuropathy in a dialysis patient unresponsive to IVIG. The Question Interpreter generated queries targeting treatment effectiveness rather than diagnostic patterns (e.g., "role of kidney transplant for recovery in IVIG-nonresponsive uremic neuropathy"). The Research Retrieval agent gathered evidence on transplantation outcomes, including the critical distinction that "renal transplantation results in significant clinical recovery" in uremic

neuropathy but "may have little effect in diabetic patients with end-stage renal disease." The Answer Synthesis agent systematically evaluated each option, selecting kidney transplantation based on its potential to correct the underlying metabolic derangements rather than providing symptomatic management. The Validator confirmed this reasoning by verifying that clinical features matched uremic rather than inflammatory neuropathy, approving kidney transplantation as addressing the root cause.“

COMMENT: 5. Figure quality and consistency: Please improve readability of the radar plots and line plots in Fig. 3 (larger fonts, consistent color mapping across Base/RAG/Agents; align subspecialty labels; ensure ≥300 dpi for production). Add consistent axis/legend labeling and abbreviations (FKD/CCI/RC) everywhere.

RESPONSE: Fixed.

COMMENT: 6. Statistical reporting: Alongside p-values, report 95% CIs for accuracies and major deltas. If multiple comparisons are extensive, note that p-values are unadjusted exploratory or apply a simple correction (state which).

RESPONSE:

We thank the reviewer for this suggestion to improve our statistical reporting.

We added 95% confidence intervals (Wilson score method) for all accuracy measurements in Table 1 and S4 Table (Supplement 1), and throughout the Results section. For example, the LLaMA 3.3-70B-based agentic system achieved 89.2% accuracy (95% CI: 85.2-92.4%) compared to 69.5% (95% CI: 64.1-74.6%) for the base model.

We revised the Statistical Analysis section in Methods to explicitly state that reported p-values are unadjusted for multiple comparisons. Given the exploratory nature of our evaluation across multiple models and implementation approaches (13 pairwise comparisons total: 10 base-vs-RAG and 3 base-vs-agents), we report unadjusted p-values with a nominal $\alpha=0.05$ threshold. We acknowledge that these findings should be interpreted as hypothesis-generating, with the primary evidence for our framework's effectiveness coming from the consistency of improvements across models, complexity levels, and subspecialties rather than from individual p-values.

COMMENT: 7. Data/code and rights: You state all data are in supplements and code is on GitHub. For PLOS, please:

• Provide stable DOIs/archival links (e.g., Zenodo/OSF) for code, prompts, and per-question annotations/complexity labels.

• Confirm permission to share translated board questions or, if rights restrict verbatim text, provide de-identified/paraphrased stems sufficient to reproduce results plus answer keys.

• Document seeds, model versions, embedding model, chunking (you note 512/128), and retrieval settings to ensure full reproducibility.

RESPONSE:

We thank the reviewer for these important suggestions to ensure data accessibility and reproducibility.

Code and data archival: We have deposited all code, prompts, and per-question annotations (including complexity labels) in a permanent archival repository with a stable DOI: 10.5281/zenodo.17329597. The GitHub repository (https://github.com/moransorka1/neurological-reasoning-benchmark) remains available for active development and community contributions.

Board certification questions: The Israeli Board Certification Exams in Neurology are publicly available on the Israeli Medical Association website (https://www.ima.org.il/internship/Exams.aspx), where examination materials and answer keys are provided for educational purposes. We found no explicit copyright restrictions or terms of use prohibiting academic use or translation of these materials. The complete dataset with English translations of all 305 questions, answer keys, and complexity classifications is available in Supplement 2.

Reproducibility details: We expanded the "Experiments Evaluation" subsection in the Methods section to include comprehensive reproducibility specifications:

> *"We evaluated multiple commercial and open-source LLMs using standardized prompts (temperature 0.7, top-p 0.9). Models spanned three categories: general-purpose (OpenAI GPT-4 family, LLaMA), reasoning-specialized (OpenAI-o1, DeepSeek R1), and medical-domain (OpenBioLLM, MedLLaMA3, Meditron), with parameter scales from 8B to 70B. Model versions were: OpenAI o1-preview (2024-12-01-preview), GPT-4o and GPT-4o-mini (2024-08-01-preview), LLaMA 3.3-70B-Instruct (December 6, 2024), LLaMA 3.1-8B-Instruct (July 23, 2024), DeepSeek-R1-Distill-Llama-70B and DeepSeek-R1-Distill-Llama-8B (January 2025), Meditron-70B, OpenBioLLM-70B, and MedLLaMA3 v20. Models spanned three categories: general-purpose (OpenAI GPT-4 family, LLaMA), reasoning-specialized (OpenAI-o1, DeepSeek R1), and medical-domain (OpenBioLLM, MedLLaMA3, Meditron), with parameter scales from 8B to 70B.*
>
> *Our RAG framework used Bradley and Daroff's Neurology in Clinical Practice (8th edition, 2022) [**Error! Reference source not found.**] as the knowledge base, processed through semantic chunking (512 tokens with 128 overlap) and embedded using BAAI's bge-large-en-v1.5 model (normalized embeddings, batch size 32). The vector database (ChromaDB version 0.5.10) retrieved the top 60 most similar passages per query using cosine similarity. After RAG evaluation, we selected top-performing models (o1, GPT-4o, LLaMA-70B) for testing within our agentic framework."*

Reviewer #1:

COMMENT: This manuscript addresses an important question in clinical AI: how to overcome reasoning limitations in LLMs, especially in neurology, a domain requiring high cognitive complexity. The authors present a compelling benchmark and a novel multi-agent system that convincingly improves performance across complexity tiers and neurological subspecialties. Strengths include:

- A robust, multi-dimensional complexity framework applied to 305 real exam questions.
- Transparent benchmarking across base, RAG-enhanced, and agentic approaches.
- Use of both Israeli board exams and MedQA data to establish generalizability.
- The multi-agent framework is especially commendable for emulating specialized cognitive processes, which is a promising direction for clinical decision support systems.

RESPONSE: We are grateful for your thoughtful review and for recognizing the clinical significance of addressing LLM reasoning limitations in neurologically complex domains. Your recognition of the multi-dimensional complexity framework and the systematic evaluation across implementation approaches is encouraging, as these were core design decisions intended to provide rigorous assessment of AI capabilities in specialized medical reasoning.

COMMENT: Clarify why specific hyperparameters (e.g., temperature 0.7, top-p 0.9) were chosen for all models and whether any tuning was performed.

RESPONSE: See editor comment 2

COMMENT: Expand briefly on error analysis -- particularly cases where even the top-performing agentic system failed (e.g., were these ambiguous, underspecified, or low-consensus items?).

RESPONSE: See editor comment 3

COMMENT: Consider revising some figures (e.g., radar plots) for improved readability and add labeling consistency.

Proofread to eliminate small typographical errors and reduce redundancy in language.

Overall, this is an excellent, impactful study that I recommend for publication following minor revisions.

RESPONSE:

Fixed.

<u>Reviewer #2:</u>

COMMENT: Thank you for an interesting topic explaining the application for LLM's in a medical setting. You have provided a good review of LLM' and how your multi-agent system achved better results.

RESPONSE: Thank you for your review and positive feedback on the work.

COMMENT: Can you clarify how does your LLM approach address the neurological clinical reasoning aspect? (i.e. what disease or condition does a patient have when applied). What about the neurological therapeutic reasoning aspect? (i.e. how your approach treats the condition after diagnosing the condition).

It would be worth making it clearer if you have considered this aspect in your paper.

RESPONSE:

Reviewer #3:

COMMENT: 1. The paper presents that RAG "had limited effectiveness on the toughest questions" and the improvement was "often modest". But the improvement for GPT-4o is a stellar leap from 80.5% to 87.3%, which is a healthy gain. It seems to be in contravention of the "modest" tag. The authors must clear this to prevent confusion

RESPONSE:

We thank the reviewer for identifying this important inconsistency in our characterization of RAG's effectiveness. We have revised the manuscript to more accurately reflect that RAG benefits were inversely related to base model performance:

In the **Abstract**, we changed "RAG provided modest benefits but limited effectiveness on complex reasoning questions" to "RAG provided variable benefits across models; substantial improvements for mid-tier models like GPT-4o (80.5% to 87.3%) and smaller models, but limited effectiveness on the highest complexity questions regardless of model size."

In the **Results section**, we replaced the opening sentence "The RAG enhancement demonstrated improvements for some models, though these gains were often modest" with "The RAG enhancement demonstrated variable effectiveness across models, with benefits inversely related to base model performance." We now explicitly characterize the different levels of improvement (dramatic for smaller models, substantial for mid-tier, minimal for top-tier) and clarify that the "limited effectiveness" specifically refers to Level 3 complexity questions across all models, not to overall RAG performance.

In the **Discussion section**, we updated our summary to reflect the full range of RAG improvements rather than characterizing them uniformly as "modest."

COMMENT: 2. The decline in performance of LLAMA 3.3-70B and OpenA-01 when applying RAG on MedQA dataset is an important finding that is poorly described in the abstract or main results. While the discussion section illuminates one of the potential reasons (the knowledge base of RAG being trained on board questions, not MedQA), it needs to be highlighted much earlier for better comprehension.

RESPONSE: See editor comment 1

COMMENT: 3. The authors need to revise RAG's description of performance so that it is a better reflection of numerical results. While a few of the models had a small gain, the 6.8 percentage point increase for GPT-4o and the 21.1 percentage point increase for DeepSeek-R1-8B are not "modest." Use more precise language, e.g., "RAG's performance varied significantly across models with substantial gains for some but only minimal improvement for others."

RESPONSE:

We thank the reviewer for this valuable suggestion. We revised the manuscript to ensure that RAG's performance description accurately reflects the quantitative results and model-specific variability. Specifically, we replaced uniform characterizations such as "modest" with precise, data-supported language emphasizing that RAG's impact varied substantially across models.

COMMENT: 4. The authors need to mention a brief note in the RAG Framework Enhancement subsection to explain why the MedQA dataset performance decline took place. Citing the point that the RAG knowledge base was tailored for board questions and less so for MedQA would be important.

RESPONSE: See editor comment 1

COMMENT: 5. The article can be strengthened with the addition of a citation that would support the effectiveness of AI in addressing intricate medical issues, particularly those related to multi-stage reasoning or data fusion. The suggested reference article "AI and Blockchain in Finance: Opportunities and Challenges for the Banking Sector", is an ideal option. The article describes how an amalgamation of AI and blockchain can enhance risk management and operational efficiency in finance, which is a comparable industry with intricate data integration and security requirements. This citation would help to support the academic foundation of the paper by illustrating how AI's application in other data-intensive complex industries validates the authors' arguments on its use in neurology.

RESPONSE:

We thank the reviewer for the suggestion to strengthen our discussion of AI's effectiveness in addressing intricate multi-step reasoning tasks across different domains.

We expanded the Introduction section to include additional context on multi-stage reasoning and challenges beyond medicine. We added references to recent work demonstrating that LLMs struggle with complex multi-step tasks across domains: Goodell et al. [19] showed that equipping LLMs with specialized medical calculation tools (an agentic approach) reduced calculation errors by >10× compared to un-augmented models, demonstrating the value of structured approaches for complex medical reasoning. We also incorporated references to multi-step reasoning benchmarks in finance, including the FinBen benchmark [21], which found that

even top LLMs "struggle with advanced reasoning and complex tasks" requiring multi-stage analysis, and DABstep [22], which comprises hundreds of multi-stage financial data challenges.

COMMENT: 6. I recommend that the authors revise the recommendation changes and suggestions to add and proceed with the further review process for publication.

RESPONSE: Thanks!
